# Safety Outcomes and Related Tolerability and Biological Responses of Vibration‐Assisted Orthodontic Tooth Movement: A Harm‐Focused Systematic Review of RCTs

**DOI:** 10.1155/ijod/7774426

**Published:** 2026-02-18

**Authors:** Mohamad Radwan Sirri, Mohammad Osama Namera, Mohamad Yaman Salahi Alasbahi, Zaher Alswaidan

**Affiliations:** ^1^ Department of Orthodontics, Faculty of Dentistry, University of Damascus, Fayez Mansour Street Al-Mazzah, Damascus, 12110, Syria, damascusuniversity.edu.sy; ^2^ Department of Restorative Dentistry and Endodontics, Faculty of Dentistry, University of Damascus, Fayez Mansour Street Al-Mazzah, Damascus, 12110, Syria, damascusuniversity.edu.sy

**Keywords:** adverse effects, GRADE, orthodontic pain, randomized controlled trial, root resorption, safety, systematic review, vibration-assisted orthodontic tooth movement

## Abstract

Vibration‐assisted orthodontic tooth movement (VA‐OTM) has been promoted as an adjunct with the potential to accelerate treatment, yet its safety profile—together with related tolerability and biological responses—remains uncertain. This harm‐focused systematic review of randomized controlled trials (RCTs) primarily appraised clinical safety end points of VA‐OTM, while explicitly distinguishing these from tolerability/acceptability outcomes and biological response/surrogate markers. The protocol was prospectively registered in PROSPERO (CRD420251166672). Reporting followed the PRISMA‐Harms extension and Cochrane guidance for harms. Major databases and gray literature were searched through August 2025 without restrictions. Eligible RCTs compared intraoral vibration with no adjunct or sham during fixed‐appliance or clear‐aligner therapy. Outcome‐level risk of bias was assessed using RoB 2 and certainty of evidence with Grading of Recommendations Assessment, Development, and Evaluation (GRADE); heterogeneity precluded meta‐analysis, so synthesis was narrative. Twenty‐three RCTs (902 participants) were included. For clinical safety end points, root resorption during canine retraction, including severe resorption (>2 mm), was similar between groups, although one of two premolar tipping studies reported smaller resorption crater volumes with vibration (*p* = 0.003). Periodontal indices and tooth mobility generally did not differ between groups (*p* > 0.05). For tolerability/acceptability outcomes, vibration did not consistently reduce pain. During fixed‐appliance alignment, one RCT reported lower pain scores with vibration (~1.0–2.3/10 vs. 4.5–6.8/10; *p* < 0.001), whereas four RCTs showed no significant difference (*p* > 0.05). In clear‐aligner therapy, two RCTs showed early reductions in pain (*p* < 0.05; *p* = 0.006) and two did not (*p* > 0.05). Oral health–related quality of life and analgesic use showed no clear benefit (*p* > 0.05). Biomarker findings were inconsistent: four RCTs reported higher IL‐1β and/or PGE_2_ levels with vibration (*p* = 0.001, *p* < 0.05, *p* ≤ 0.03, and *p* < 0.001), whereas three found no meaningful change. Most outcome‐level RoB 2 judgments raised some concerns (about 89%), and the certainty of evidence was low to very low for most outcomes. Within these limitations and short follow‐up, adjunctive low‐intensity intraoral vibration appears unlikely to compromise root integrity, periodontal health, or tooth stability, but does not justify routine use primarily for pain control, quality‐of‐life improvement, or root‐resorption prevention; larger, long‐term harm‐focused RCTs with standardized safety outcomes are needed before extrapolating findings to untested devices or dosing regimens.

**Summary**

• A priori safety was defined using a clinical/regulatory framework (device‐related adverse events and clinically meaningful tissue deterioration).
• Outcomes were classified into clinical safety end points, tolerability/acceptability outcomes, and biological response (surrogate) markers.
• Across 23 randomized controlled trials (RCTs; 902 participants), vibration did not show clinically meaningful worsening of root integrity or periodontal clinical status.
• Pain effects were small, inconsistent, and often limited to early time points without consistent clinical relevance.
• Biomarker changes (e.g., IL‐1β and PGE_2_) were heterogeneous and should be interpreted as mechanistic responses, not clinical harm.

## 1. Introduction

Since the beginning of modern orthodontics, the central premise has been that “mechanics reshape biology” [1]. Orthodontic forces applied to the dentition are transduced by the supporting tissues into cellular signals that trigger alveolar bone remodeling and reorganization of the periodontal ligament (PDL) [2]. Within this framework, vibration has emerged as an adjunctive mechanical stimulus, commonly referred to as vibration‐assisted orthodontic tooth movement (VA‐OTM) [2]. It uses low‐magnitude, high‐frequency impulses to activate mechanobiological pathways without increasing the magnitude of the clinical orthodontic forces [3].

Historically, the concept of VA‐OTM has been informed by research on low‐magnitude, high‐frequency vibration (LMHFV) in bone. Laboratory and animal studies have shown that mild cyclic stimulation can influence bone cell activity and differentiation [4]. These findings suggest that VA‐OTM may modulate force‐loaded alveolar tissues through mechanotransduction pathways that regulate bone remodeling during tooth movement [5].

Biologically, the PDL functions as a mechanosensor that converts mechanical impulses into molecular signals via integrins, focal adhesion complexes, and stretch‐activated ion channels [6]. In particular, the mechanosensitive channel PIEZO1 is activated by orthodontic forces and plays a key role in regulating alveolar bone remodeling during tooth movement [6].

This process engages the central RANK/RANKL/OPG molecular pathway of tooth movement. Modulation of this pathway enhances osteoclast differentiation on the compression side while reducing its inhibitor, OPG, thereby shifting the balance between bone resorption and formation. In parallel, osteoblasts are activated on the tension side. The coordinated action of these mechanisms permits tooth movement through organized remodeling of the alveolar bone [7].

From a technical standpoint, vibration is typically delivered by at‐home intraoral devices used for approximately 20 min per day at a frequency of around 30 Hz. These devices generate an oscillatory force of about 25 g. This “microdose” of vibration is intended to stimulate mechanobiological signaling pathways without increasing the orthodontic force prescribed by the clinician [8].

The expected effects of vibration as a low‐intensity mechanical modulator arise from three interrelated factors [3]: (1) enhancement of alveolar bone remodeling through early activation of osteoclastogenesis followed by later osteoblastogenesis, mediated by RANKL/OPG signaling and mechanically responsive cytokines; (2) modulation of periodontal stem cell behavior and regulation of fiber and collagen formation in response to cyclic shear; (3) variable impacts on patient experience, including pain and discomfort, which depend on pulse frequency, dose, and treatment adherence [3].

Clinically, randomized controlled trials (RCTs) have examined both the putative benefits and potential adverse effects of vibration as primary or secondary outcomes. These studies have evaluated pain and discomfort [9, 10], root resorption [11, 12], periodontal indices [13], molecular biomarkers [10, 14], and changes in tooth mobility [15]. The magnitude and direction of these effects appear to depend on tissue characteristics, dose–time parameters, and the degree of alignment between vibration and orthodontic loading. This may explain why recent clinical trials have produced mixed findings: some report significant advantages of VA‐OTM [9, 11, 16], whereas others show no clinically relevant differences [10, 12–14]. Accordingly, vibration is better conceptualized not as an additional force, but as a signaling modulator that targets key mechanobiological pathways in the PDL and alveolar bone to promote more efficient remodeling with lower mechanical stress [3].

Although there has been growing interest in using VA‐OTM with the potential aim of accelerating treatment [17], its evaluation requires a clear conceptual safety framework that distinguishes between true clinical harm, tolerability/acceptability, and biological response. Safety, in the context of device‐based interventions, is defined as: (1) the absence of device‐related adverse events/adverse effects according to medical‐device clinical investigation terminology and (2) the absence of clinically meaningful deterioration in hard or soft tissues beyond what is expected during conventional orthodontic tooth movement, in line with standard and regulatory frameworks for medical‐device safety reporting [18–20]. Accordingly, outcomes such as root resorption and clinically meaningful deterioration of periodontal tissues are considered key clinical safety indicators [21, 22]. In contrast, patient‐reported pain/discomfort is an indicator of tolerability [23], while biomarkers primarily reflect mechanistic or surrogate biological responses rather than adverse events per se [24].

Despite the concerns raised by individual RCTs, systematic reviews that primarily and rigorously appraise clinical safety outcomes of VA‐OTM—while explicitly distinguishing these from tolerability/acceptability measures and surrogate biological responses—remain scarce. Most existing reviews focus on the efficacy of VA‐OTM in shortening treatment duration [3, 25, 26], often giving less weight to harms and safety outcomes, and sometimes aggregating outcomes such as pain/discomfort and biomarkers under “safety” without a clear outcome‐classification framework. Several reviews also include controlled clinical trials (CCTs) and animal studies, which can dilute the overall strength and directness of the clinical evidence [3, 26]. In addition, some reviews assess risk of bias only at the study level rather than at the outcome level [27], a method discouraged by the Cochrane collaboration because it may obscure outcome‐specific biases [28]. Harms reporting is also prone to bias, as safety outcomes may receive less emphasis than tooth movement rate, and key methodological aspects—such as blinding, handling of missing data, and intention‐to‐treat analyses—are often inadequately reported.

These gaps highlight the need for a harm‐focused systematic review anchored in an explicit framework that distinguishes clinical safety end points from tolerability outcomes and surrogate biological responses. Prioritizing RCTs, outcome‐level RoB 2, and transparent Grading of Recommendations Assessment, Development, and Evaluation (GRADE) judgments with prespecified harms can provide clinically interpretable evidence to guide balanced, evidence‐based use of VA‐OTM.

## 2. Materials and Methods

### 2.1. Scoping Search

A preliminary scoping search was conducted in PubMed and Scopus to identify prior systematic reviews focusing on the safety and adverse effects of VA‐OTM. No comprehensive, safety‐focused reviews restricted to RCTs were identified. The review protocol was prospectively registered in PROSPERO (CRD420251166672). Reporting followed the PRISMA‐Harms extension [29]; a completed PRISMA‐Harms checklist with page/line mapping is provided in Supporting Information 1: Table S1. The review also followed the Cochrane handbook guidance on the evaluation and reporting of adverse effects [30]. Insights from this scoping phase informed the choice of databases, search terms, and prespecified safety outcomes in the present review.

### 2.2. Question and Eligibility Criteria

#### 2.2.1. Research Question

In RCTs, does VA‐OTM, compared with no adjunct or sham, increase device‐related adverse events or clinically meaningful deterioration in dental/periodontal tissues (clinical safety end points), and how does it influence tolerability (patient‐reported symptoms) and biological response markers?

#### 2.2.2. Operational Definition and Outcome Framework

For this review, safety was operationally defined as the absence of device‐related adverse events/adverse effects and the absence of clinically meaningful hard‐ or soft‐tissue deterioration beyond what is expected during conventional orthodontic tooth movement [18–20], consistent with medical‐device clinical investigation terminology and regulatory concepts for adverse‐event reporting (including serious and/or unanticipated events when available). This operational definition is consistent with international medical‐device standards for good clinical practice and risk management in device investigations (e.g., ISO 14155:2020 and ISO 14971:2019) [31] and with U.S. FDA IDE safety‐reporting terminology (including unanticipated adverse device effects) [19]. Accordingly, and to ensure alignment between the research question and analyzable end points, outcomes were prespecified and categorized into three domains: (1) clinical safety end points, including root resorption and clinically meaningful gingival/periodontal supporting‐tissue deterioration, with periodontal deterioration interpreted using recognized standardized thresholds (e.g., bleeding on probing [BOP] ≥ 10% as a commonly used cutoff for gingival inflammation, and pocketing/clinical measurement criteria when reported in the trials) [21, 22]; (2) tolerability/acceptability outcomes, such as pain, analgesic use, and oral health–related quality of life [23]; (3) biological response/surrogate markers (e.g., inflammatory mediators), treated as mechanistic indicators that do not constitute adverse events per se unless linked to clinically important harm [24]. Harms data were extracted and reported in accordance with methodological guidance for addressing harms in systematic reviews.

#### 2.2.3. Eligibility

Eligibility criteria were prespecified using the PICOS framework (Population, Intervention, Comparison, Outcomes, Study design; Table 1).

**Table 1:** PICOS framework and the searched electronic databases.

Participants	Healthy individuals of any age, sex, or ethnicity receiving active orthodontic treatment (fixed appliances or clear aligners), with or without extraction plans; no restrictions on malocclusion type or treatment indication
Interventions	Vibration‐assisted acceleration delivered by any extra‐ or intraoral device, frequency, or regimen
Comparisons	No adjunct, usual care, or sham/placebo vibration
Outcomes	Safety and adverse effects, including patient‐reported outcomes (PROs; pain, discomfort, oral health quality of life [OHIP‐14], and analgesic use), structure and safety (root resorption), biomarkers, periodontal and clinical indices (periodontal parameters indices and tooth mobility)
Study design	Human randomized controlled trials only, using either parallel‐group or split‐mouth designs. With a sham/no‐adjunct comparator and evaluable safety data

#### 2.2.4. Exclusions

The following were excluded: nonrandomized designs (e.g., cohort, case–control, or cross‐sectional studies), quasi‐randomized trials, case series, and case reports; animal or in vitro studies; narrative reviews, editorials, and expert opinions; conference abstracts without sufficient data; RCTs with unusable or incomplete safety data (e.g., incomplete outcome reporting or unavailable full text).

### 2.3. Search Strategy

A systematic search was carried out by two independent reviewers (Mohamad Radwan Sirri and Mohammad Osama Namera) through August 2025 in PubMed, Embase, Scopus, Web of Science, and the Cochrane Central Register of Controlled Trials (CENTRAL), with additional searches in the Trip database and Google Scholar. Gray literature was explored via OpenAIRE and EBSCO Open Dissertations. No language, date, or publication status restrictions were imposed. Hand searching included the reference lists of eligible articles and four core orthodontic journals: American Journal of Orthodontics and Dentofacial Orthopedics (AJODO), European Journal of Orthodontics (EJO), Journal of Orthodontics, and Orthodontics & Craniofacial Research. The search strategy combined controlled vocabulary and free‐text terms related to orthodontics, vibration, acceleration, and safety or adverse events. Full electronic search strategies are provided in Supporting Information 2: Table S2.

### 2.4. Study Selection and Data Extraction

Two reviewers (Mohamad Radwan Sirri and Mohammad Osama Namera) independently screened titles and abstracts, removed duplicates, and retrieved full texts for potentially eligible studies. Screening was performed in two stages (title/abstract and then full text), and reasons for exclusion at the full‐text stage were documented. Any disagreements were resolved through consultation with a third reviewer (Mohamad Yaman Salahi Alasbahi).

Data extraction was performed independently and in duplicate using a piloted, standardized extraction form. Extracted items included study characteristics, participant and appliance details, vibration parameters (frequency, magnitude, and daily exposure time), comparator type, follow‐up duration, and all reported safety, adverse‐event, and patient‐reported outcomes. Data were initially extracted by one reviewer (Zaher Alswaidan) and independently verified by a second reviewer (Mohamad Yaman Salahi Alasbahi).

### 2.5. Clinical Relevance Criteria

Clinical relevance was prespecified for each outcome domain (resorption, periodontal indices, tooth mobility, pain/discomfort, OHIP‐14, analgesic use, and biomarkers) and interpreted based on magnitude, consistency across time points, and concordance with patient‐important and tissue‐level clinical end points rather than statistical significance alone.

For external apical root resorption (EARR), clinical relevance was judged primarily using clinically relevant/severe thresholds (≥2 mm) and severity grading approaches (e.g., Malmgren‐type categories), emphasizing whether vibration altered the frequency of clinically relevant/severe resorption rather than only mean differences [32]. Periodontal clinical relevance was judged using accepted periodontal health/gingivitis definitions (e.g., gingival health typically corresponding to BOP < 10% with shallow probing depths [PDs], where reported) and by the emergence/worsening of PD/bleeding patterns suggestive of inflammation [21]. Tooth mobility assessed by periotest was considered clinically relevant only when changes exceeded expected device measurement error (manufacturer‐reported error up to approximately ±2 periotest value (PTV) in anterior teeth and ±3 PTV in posterior teeth) and were directionally consistent with other clinical safety end points [33].

For pain/discomfort measured on a 0–100 mm visual analog scale (VAS), between‐group differences were considered potentially clinically important only if they approached or exceeded approximately 10–12 mm, consistent with published minimum clinically important/significant differences on a 100 mm VAS [34, 35]. For pain measured on a 0–10 numeric rating scale (NRS), clinically meaningful differences were interpreted using adolescent/clinical interpretability evidence suggesting that approximately 1 point (and/or ~12.5% change) represents a minimally clinically significant difference, while also considering whether changes corresponded to patient‐anchored outcomes such as perceived need for analgesia [36, 37]. Analgesic use (proportion requiring rescue medication and/or number of doses, when reported) was treated as a patient‐important anchor of tolerability and interpreted alongside pain trajectories rather than in isolation [37]. For oral health–related quality of life (OHIP‐14), clinical relevance was contextualized against orthodontic MID estimates (reported to be large in magnitude in available orthodontic MID work) and interpreted cautiously when baseline OHIP‐14 values suggested floor effects [38].

Finally, biomarker changes (e.g., IL‐1β, PGE_2_, and RANKL/OPG) were interpreted as surrogate/mechanistic signals; therefore, isolated statistically significant biomarker shifts were not construed as clinical harm unless accompanied by concordant clinically meaningful worsening in pain burden, EARR severity, periodontal indices, or mobility, consistent with established definitions cautioning that surrogate end points do not directly measure how patients feel or function [24, 32].

### 2.6. Risk of Bias Assessment

Outcome‐level risk of bias was assessed using the Cochrane RoB 2 tool [39] by two independent reviewers (Mohamad Radwan Sirri and Mohammad Osama Namera), with disagreements adjudicated by a third reviewer (Mohamad Yaman Salahi Alasbahi). To avoid the study‐level masking effect noted by Cochrane [28], variable‐level judgments were reported. Domain‐level decisions are summarized in Supporting Information 3: Table S3.

### 2.7. Certainty of Evidence (GRADE)

The certainty of evidence for adverse events and other safety outcomes in RCTs comparing VA‐OTM with no adjunctive intervention or sham was appraised using the GRADE approach [40]. Outcome‐specific ratings (high, moderate, low, or very low) were based on considerations of risk of bias, inconsistency, indirectness, imprecision, and publication bias. Two authors (Mohamad Radwan Sirri and Mohammad Osama Namera) conducted independent GRADE assessments, and any discrepancies were resolved by a third reviewer (Mohamad Yaman Salahi Alasbahi).

### 2.8. Summary Measures and Approach to Synthesis

Because the included studies exhibited substantial clinical and methodological heterogeneity, a quantitative meta‐analysis and formal assessment of publication bias were not performed. Instead, the evidence was synthesized narratively using a structured approach. Outcomes were grouped according to the type of vibration intervention, the nature of the comparator, and the duration of follow‐up. Key similarities and differences in study design, participant characteristics, and outcome definitions were described systematically and transparently.

## 3. Results

### 3.1. The Search Flow and the Retrieved Studies

**Figure 1:**
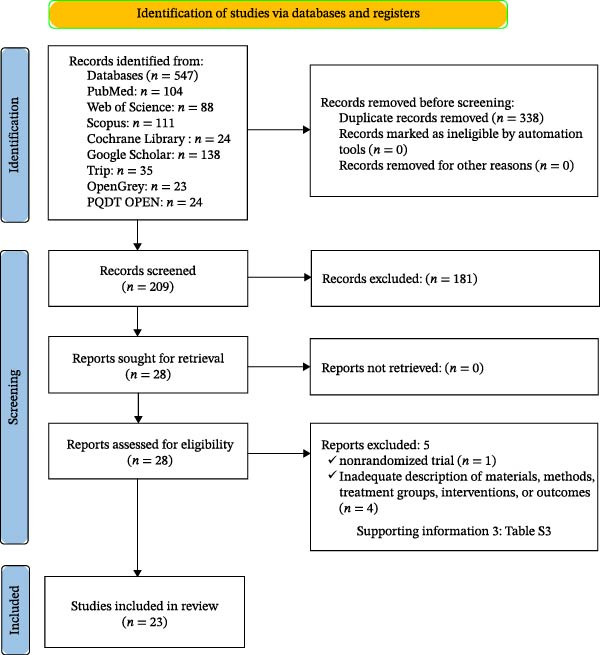
Preferred Reporting Items for Systematic Reviews and Meta‐Analyses (PRISMA) flow diagram of the included studies.

The electronic search identified a total of 547 records. After removing duplicates, 338 records remained for title and abstract screening. Based on the prespecified inclusion and exclusion criteria, 181 were excluded. The full texts of the remaining 28 reports were assessed in detail, and five were excluded with documented reasons (Supporting Information 4: Table S4). In total, 23 studies met the eligibility criteria and were included in this systematic review. The selection process and reasons for exclusion are summarized in the PRISMA flow diagram (Figure 1).

### 3.2. Characteristics of the Included Studies

Twenty‐three RCTs involving 902 participants were included. The trials evaluated vibration as an adjunct in four orthodontic contexts: canine retraction (*n* = 6 RCTs [12, 13, 41–44]), leveling and alignment (*n* = 14 RCTs [9, 10, 14–16, 45–53]), en masse retraction (*n* = 1 RCT [54]), and experimental buccal tipping (*n* = 2 RCTs [11, 55]). Safety and adverse effects were organized into three domains and seven variables:

1. Clinical safety end points: Hard‐tissue safety (EARR) [11, 12, 41, 53, 55], and periodontal and clinical status (periodontal indices [10, 13] and tooth mobility [15]).
2. Tolerability/acceptability outcomes: Patient‐reported pain/discomfort [9, 10, 12, 13, 15, 16, 41, 42, 45–52], oral health‐related quality of life using the OHIP‐14 instrument [15, 51], and analgesic use [16, 48, 50].
3. Biological response/surrogate markers: Biomarkers reflecting biologic response [10, 14, 15, 43, 44, 47, 54].

The main characteristics of the included studies are summarized in Table 2.

**Table 2:** Characteristics of included RCTs in the systematic review.

Study setting				Orthodontic parameters			Physiologically accelerated intervention specifics			Outcomes studied		Results
Author/ country/ design	Treatment comparison	Sample size, *N* (F/M), ages (years), mean	Treatment goal	Appliance characteristics	Extractions (yes/no)	Anchorage type	Intervention device specifications daily usage	Follow‐up	Duration	Outcome type	Measurement method	
Abd ElMotaleb et al. [41], Egypt, parallel	2 arms: (1) AcceleDent + fixed appliance (2) Control (fixed appliance only)	*N* = 32 (F/M = 32/0) = 64 canines, age 15–21 years, mean: not reported	Canine retraction	MBT 0.022 × 0.028; alignment up to 0.016 × 0.022 SS → base arch 16 × 22 SS; NiTi closing spring 150 g	Upper first premolars bilaterally; after leveling and alignment and before retraction	Buccal TADs 1.8 × 8 mm between U5 and U6; indirect anchorage with 0.019 × 0.025 SS	AcceleDent Aura: ~0.25 N at 30 Hz; 20 min/day; adherence ~89%	Every 4 weeks; recalibrate 150 g; monthly models T0–T4	4 months (retraction phase)	Primary: Rate/total distance of canine retraction (monthly and cumulative) from 3D models. Secondary: CBCT metrics (canine crown/root displacement, tipping, torque, rotation; anchorage loss), pain (VAS), root length/resorption	3D models: Scanned casts (3Shape/OrthoAnalyzer); superimposition on the third palatal rugae; linear distances in mm; color‐map check; blinded assessor. CBCT: InVivo; voxel ~0.3 mm; reference planes (MSP/HP/FP); linear (mm) and angular (°) measures. Pain: VAS 0–10 at 0, 24, 72 h, and 7 days after activation	No significant overall acceleration; month − 3 difference only (favor intervention). CBCT, pain, root length, anchorage: NS. Conclusion: the device did not speed movement or reduce pain
Alansari et al. [47], USA and Colombia, parallel	5 arms, five groups: (1) 14‐day control (no vibration) (2) 7‐day sham (no vibration) (3) 7‐day vibration (VPro5) (4) 5‐day sham (discontinued) (5) 5‐day vibration (VPro5)	Enrolled 75 (planned); analyzed completers: control 13, 7‐sham 13, 7‐vibration 14, 5‐vibration 13; 5‐sham 0 (stopped), age range: 18–45 years, group means ~25–32 years, overall mean not reported	Alignment and leveling	Invisalign (SmartTrack). Target movement per aligner: 0.25 mm AP on one lower incisor; aligner wear 22 h/day; only tipping movement assessed; no attachments/IPR during study	No	N/A (clear aligners; no TADs)	VPro5 high‐frequency acceleration: 120 Hz, 0.03 g; used with aligners seated. 5 min/day, preferably before sleep or during the longest continuous wear. Compliance is logged daily	Baseline and end‐of‐study scans; GCF at end of 2nd aligner; office visits aligned with aligner‐change intervals (14/7/5 days), with compliance reviewed each visit	4 aligners per participant—56 days (14‐day control), 28 days (7‐day sham/vibration), 20 days (5‐day vibration; 5‐day sham discontinued)	Primary: % tracking vs. ClinCheck prediction. Secondary: Pain (0–10 NRS on Days 1 and 3), and GCF cytokines/bone‐remodeling markers	Tracking: iTero scans superimposed to ClinCheck using “Automated Superimposition—Best Fit”; 300% magnification; software error <0.2 mm; blinded assessors. Pain: 0–10 numeric rating scale (Days 1 and 3). GCF: MILLIPLEX/Luminex assay per manufacturer protocol	Tracking (%; mean ± SD): control 84 ± 13; 7‐sham 70 ± 16; 7‐vibration 90 ± 14 (vs. 7‐sham *p* = 0.003); 5‐vibration 84 ± 12; 5‐sham discontinued due to nontracking. Pain: 7‐vibration lower than 7‐sham Day 1 (*p* < 0.020) and Day 3 (*p* < 0.026), and lower than control Day 1 (*p* < 0.034). GCF: significant increases of cytokines/bone markers with vibration vs. control/sham. Conclusion: VPro5 for 5 min/day enables 7‐ or 5‐day aligner changes without loss of tracking and reduces early pain
Azeem et al. [13], Pakistan and Saudi Arabia, SMD	2 arms, within‐subject: (1) Electric‐toothbrush vibration side (2) Nonvibration control side during maxillary canine retraction	*N* = 28 patients (F/M = 18/10), age 18–24 years, mean 20.8 years	Canine retraction	MBT 0.022‐in (3M gemini); initial 0.014/0.016‐in NiTi; then 0.019 × 0.025‐in TMA (2 months); segmental 0.020‐in SS; NiTi closed‐coil (GAC) 100 g from molar band hook to canine hook; no reactivation at R2/R3	Yes—bilateral upper first premolars; 3‐month consolidation before experiment	Conventional molar anchorage with segmental mechanics; no TADs	Oscillating‐rotating electric toothbrush (Oral‐B Triumph, OD17 ortho head), ~125 Hz; applied on the mesiolabial surface of the test canine 20 min/day for 60 days (after the first month of retraction); compliance diary	Monthly visits (R1, R2, and R3); batteries provided; pain diaries reviewed; plaque index recorded at R0–R3	3 months retraction (R0→R3); vibration applied during last 2 months	Primary: Amount/rate of canine movement (monthly and cumulative). Secondary: Pain (VAS) and plaque index (PI)	Primary (tooth movement): Plaster models with palatal “plug” referenced to 3rd palatal rugae; one blinded examiner; digital caliper 0.01 mm. Secondary (pain/PI): Pain diary on 100‐mm VAS (daily × 7 then weekly → monthly score); PI at six sites/tooth at R0–R3 by blinded examiner	Tooth movement: no significant difference V vs. NV (~0.80–0.84 mm/month; total R0–R3 = 2.48 ± 0.33 mm vs. 2.43 ± 0.30 mm, *p* > 0.05). Pain: change overtime NS; no rescue meds. Plaque: low and NS between sides. Conclusion: electric‐toothbrush vibration did not accelerate tooth movement
Bisht et al. [45], India, parallel	Six arms: (1) MBT + vibration (2) SLB + vibration (3) MBT control (no device) (4) SLB control, MBT (5) placebo (dummy) (6) SLB placebo (dummy)	*N* = 36 (6/group); F/M: not reported, age: not reported	Alignment and leveling	MBT twin brackets and passive self‐ligating brackets (0.022″ MBT); archwires: 0.014″ NiTi (first 6 wks) → 0.016″ NiTi (next 6 wks); SS ligatures (0.010″) for twin; nonextraction; no IPR	No (nonextraction sample)	Conventional (no TADs)	Custom vibration device: 30 Hz, 25 g; timed auto‐off at 20 min; rechargeable; bioacrylic mouthpiece 20 min/day throughout 3 months; also 1 hr after archwire placement on visit days	T0 (bonding), T1 = 6 weeks, T2 = 12 weeks; impressions/models and pain surveys at each time; no analgesics allowed	3 months (initial alignment phase)	Primary: Lower anterior alignment by Little’s Irregularity Index (LII) (T0, T1, T2). Secondary: Pain/discomfort by VAS at each visit	LII: Study models at T0, T1, and T2; measured with digital caliper (0.01 mm) by blinded assessor. Pain: 10‐cm VAS at 1 hr and 2 hr after archwire insertion at T0, T1, and T2	Alignment: Vibration significantly improved LII resolution in MBT vs. its control/placebo; no significant effect within SLB groups. Pain: Vibration significantly reduced pain at all appointments for both MBT and SLB (*p* < 0.001)
Bragassa [56], USA, parallel	3 arms: (1) 14‐day aligner wear (control) (2) 4‐day aligner wear (3) 4‐day aligner wear + vibration (AcceleDent Aura)	*N* = 33 randomized (G1 *n* = 10, G2 *n* = 12, and G3 *n* = 11); analyzed 63 arches; female ~64%, age 18–65 years, mean 32.3 years (SD = 9)	Alignment and leveling	Invisalign CAT; ≥21 active aligners; default per‐aligner movement 0.25 mm; wear 22 h/day; trios scans at visits (attachments placed per plan)	No (nonextraction CAT cohort)	N/A (aligners; no TADs)	AcceleDent Aura microvibration: ~0.25 N (~25 g) at 30 Hz (manufacturer spec used in protocol) 20 min/day (device compliance logged; aligner wear self‐reported)	T0 = the baseline visit, T4‐days, T2‐weeks, T6‐weeks, T12‐weeks (scans + pain survey each visit)	12 weeks total	Primary: % reduction in PCPDI (incisor alignment). Secondary: % accuracy of PCPDI reduction (vs. predicted). Tertiary: Pain (NRS/faces) and analgesic use; OB accuracy (exploratory)	PCPDI: digital. stl models (Trios) at T0 and T12; Geomagic DesignX; PCPDI computed for upper/lower incisors; % reduction and % accuracy per formulas; blinded assessor; Dahlberg error ~0.1 mm. Pain: Faces/NRS 0–10 at each visit; meds yes/no. Compliance: FastTrac download + self‐report	Efficiency: G1 18.9% vs. G3 29.1% (*p* = 0.003) → 4‐day+vibration more efficient than 14‐day, but G2 vs. G3 NS (*p* = 0.089) → vibration no added benefit. Accuracy: G1 48.3% vs. G2 36.1% (*p* = 0.0235) and G3 37.7% (*p* = 0.047) → accelerated schedules decrease accuracy; G2 vs. G3 NS (*p* = 0.284). Pain/meds: only one time‐point difference (G2 > G1 at 2 weeks, *p* = 0.033); meds NS; overall pain <2.5/10 and declined over time. OB: overall OB accuracy NS among groups; simple OB is more accurate than complex OB within groups. Conclusion: 4‐day wear increases efficiency but reduces accuracy; vibration does not improve efficiency, accuracy, OB, or pain
Bulic [57], USA, parallel	2 arms: (1) AcceleDent Aura + SureSmile (2) Control (SureSmile only)	Analyzed *N* = 61 (control 40: F22/M18; AcceleDent 21: F10/M11), mean age: control 14.65 ± 5.22, AcceleDent 14.49 ± 5.65 years	Alignment and leveling	MBT 0.018" twin brackets; standardized wire sequences (NiTi/Cu‐NiTi) per protocol; treatment delivered at 3 Illinois private offices with scans analyzed at UIC	No (extraction cases excluded)	Conventional fixed‐appliance anchorage (no TADs reported)	The AcceleDent Aura device was used (manufacturer’s micropulse trainer; specific force/frequency not reported in the thesis text). Protocol: 20 min twice on Day 0 and Day 1 after each adjustment, then 20 min once daily thereafter	T1 baseline (prebonding), T2 = 3 months, T3 = 6 months, T4 end of active tx or 12 months; pain: Day 0, Days 1–3 after each adjustment, then weekly until next visit	Up to 12 months of observation	Primary: Alignment (Little’s Irregularity Index).Secondary outcomes: Pain (VAS/FACES) and device compliance	Digital models from iTero; OrthoCAD measurement (plane‐to‐plane; blinded assessor). Pain via online SurveyMonkey VAS/FACES with e‐mail reminders	Alignment: No clinically meaningful between‐group differences in irregularity‐index change across intervals (larger change 0–3 mo in both groups). Pain: AcceleDent significantly lower pain at T2 (3–6 months) and T3 (6–12 months) vs. control (independent *t*‐tests, *p* = 0.006 at both time points); means: T2 1.36 vs. 2.95, T3 0.66 vs. 1.70. Compliance mean ~78%
Chouinard [58], USA, parallel	2 arms: (1) fixed appliances + AcceleDent (2) fixed appliances only (control)	*N* = 23 enrolled (AcceleDent 11 = F/M 8/3; control 12 = F/M 9/3), mean age at start: ~20.6 (vibration) and ~21.0 (control) years	Alignment and leveling	Passive self‐ligating brackets (carriere) 0.022 × 0.025, MBT; T0: 0.014″ Cu‐NiTi; T2: 0.014 × 0.025″ Cu‐NiTi; adjustments every 5–6 wks	No (nonextraction cohort; ≥5 mm mandibular crowding inclusion)	Conventional (no TADs)	AcceleDent (OrthoAccel): cyclic force = 25 g at 30 Hz; occlusal mouthpiece transmits vibration to teeth/alveolar bone. 20 min/day throughout study	T0 baseline; T1 5–6 wks; T2 10–12 wks; T3 15–18 wks	Up to 15–18 weeks (~3–4 months)	Primary: (1) Salivary biomarkers of bone remodeling; (2) lower incisor alignment rate (LII). Secondary: Tooth mobility (Periotest), pain (VAS), OHQoL (OHIP‐14)	Biomarkers: unstimulated whole saliva; ELISA (e.g., IL‐1β, IL‐8; TNF‐α below detection). Alignment: Little’s Irregularity Index on casts with a digital caliper. Mobility: Periotest on selected teeth. Pain: 0–10 VAS for 7 days postvisit. OHQoL: OHIP‐14 questionnaire	No significant differences between groups in: LII means/changes (T0→T3), pain at T0/T1/T2, tooth mobility (incisors/canines/premolars), or OHQoL; IL‐1β/IL‐8 showed no significant group differences; TNF‐α was undetectable. Recorded device compliance means ~63%
DiBiase et al. [53], UK, parallel	3 arms: (1) AcceleDent (active) + fixed appliance (2) Sham AcceleDent + fixed appliance (3) fixed‐only	Randomized *N* = 81 (<20 years). analyzed *N* = 72 (losses minimal), group ages ~14.0 years (means 13.6–14.3); sex balanced	Alignment and leveling	Standardized fixed appliances (MBT 0.022″ 3M Victory); archwire sequence: 0.014″, 0.018″, 0.018 × 0.025″ NiTi → 0.019 × 0.025″ SS; visits ~8‐weekly; no auxiliaries	Yes. Mandibular first premolars for all subjects; maxillary extraction patterns varied (4 s/5 s/mixed)	Conventional; no TADs, headgear, elastics during study period	AcceleDent intraoral vibrational device: 30 Hz, 0.2 N (occlusal wafer) 20 min/day, and 20 min before each appointment (per manufacturer guidance)	Appointments approximately every 8 weeks; LCPAs at T1 (start) and T3 (end of alignment at insertion of 0.019 × 0.025″ SS)	Mean T1→T3 = 201.6 days (95% CI 188.6–214.6)	Primary (this report): OIIRR (mm) at UR1 from T1→T3. Secondary: Proportion withsevere OIIRR >2 mm	Long‐cone periapical radiographs at T1 and T3; blinded assessor; Adobe Photoshop ruler with crown‐length correction factor; excellent repeatability (ICC high)	Mean OIIRR ~1.08 mm overall; no significant differences among active, sham, control (multivariable *Δ*Active vs. control 0.29 mm, 95% CI −0.14 to 0.72; *p* = 0.184). Severe OIIRR >2 mm: 17% overall; no between‐group differences (*p* = 0.551). Conclusion: Daily supplemental vibration did not affect OIIRR during alignment
Grove [59], Australia, SMD	2 arms: (1) Vibration (buccally directed) + 150 g orthodontic force (2) Nonvibration + 150 g orthodontic force	*N* = 14 patients/28 teeth; 11F/3M, age 12.1–15.5 years, mean 13.6 years	Experimental buccal tipping	0.022 × 0.028‐in SPEED brackets on maxillary first molar and first premolar; 0.017 × 0.025‐in TMA springs applying 150 g buccal force; lower first molar GIC bite‐buildups to minimize trauma	Yes—maxillary first premolars extracted after the 4‐week experimental period	Conventional intra‐arch anchorage (first molar–first premolar spring mechanics); no TADs reported	Oral‐B HummingBird with modified tip; buccally directed to premolar; measured ~113 Hz (~6800 RPM) (spec ~8000 RPM ~133 Hz); batteries changed every 2 weeks. 10 min/day for 28 days; compliance monitored daily by an author	Daily monitoring during the 4 weeks; end point at extraction	4 weeks (28 days)	Primary: Total root‐resorption volume per tooth. Secondary: Volume per surface (B/M/P/D) and per vertical third (cervical/middle/apical); regression of baseline resorption vs. improvement	Micro‐CT (SkyScan 1172) scanning; 60 kV, 167 µA, 360° rotation (0.23° step); 3‐D reconstruction; analysis in VGStudio	Vibration reduced total resorption volume by 0.128 mm^3^ (~33%) vs. control (mean 0.261 vs. 0.389 mm^3^; *p* = 0.003). Palatal surface significant (*p* = 0.006); mesial marginal (*p* = 0.018); apical third marginal (*p* = 0.019)
Gujar and Shivamurthy [54], India, parallel	3 arms: (1) 125 Hz vibrating electric toothbrush (2) 150 Hz vibrating electric toothbrush; (3) control (no vibrating toothbrush)	*N* = 30 (F/M = 16/14), 18–25 years, mean not reported	En masse retraction (maxillary anterior)	MBT 0.022 × 0.025 (3M Gemini); bands on upper 1st molars; initial 0.016″ NiTi → working 0.019 × 0.025″ SS; en masse retraction with elastomeric chain ~200 g	Yes—bilateral maxillary first premolars (as part of treatment)	Conventional (no TADs); friction mechanics first molar ↔ consolidated anterior segment	Custom electric toothbrush producing mechanical vibration at 125 Hz (Grp A) or 150 Hz (Grp B); applied to labial and palatal surfaces of the 6 anterior teeth 6 min/day (1 min per tooth) starting 1 day after retraction onset; standard brushing otherwise	Assessments at T0 (start retraction), T1 (30 d), T2 (60 d), and T3 (90 d); GI recorded each visit	90 days (3 months)	Primary: Rate of orthodontic tooth movement (OTM, mm/interval). Secondary: PGE2 level in GCF (pg/mL)	OTM: palatal acrylic plug indexed to 1st–3rd palatal rugae; stainless‐steel reference wires; digital caliper distance change across extraction space at T0–T3. PGE2: GCF collection (1 µL microcapillary; extracrevicular); ELISA (RayBiotech). Stats: ANOVA + Tukey (OTM); Kruskal–Wallis + Dunn (PGE2)	Both outcomes favored 150 Hz. OTM (right side, T0–T1 means): 150 Hz 1.463 ± 0.115 mm > 125 Hz 1.020 ± 0.178 mm > control 0.644 ± 0.155 mm (*p* < 0.001). Left side similar ranking (all *p* < 0.001). PGE2 (pg/mL) at T1: 150 Hz 982.8 ± 22.9 > 125 Hz 763.1 ± 121.3 > control 421.5 ± 98.2 (pairwise p ≤ 0.03); elevations persisted at T2/T3 with 150 Hz highest.conclusion: 150 Hz toothbrush vibration increased OTM rate and PGE2 vs 125 Hz and control over 3 months
Kalemaj et al. [10], Italy, parallel	3 arms: (1) CLA (conventional ligating) (2) SLA (self‐ligating) (3) SLA‐VA (self‐ligating + vibrational appliance)	*N* = 33 randomized (11/group); F/M = 19/14, mean age 13.1 ± 2.3 years (by group ~12.9, 13.3, 13.1)	Alignment and leveling	CLA: MBT twin 0.022‐in (AO). SLA: empower interactive SL (AO). standard archwire sequence: 0.014‐in thermal NiTi → 0.017 × 0.025‐in thermal NiTi → 0.019 × 0.025‐in thermal NiTi → 0.019 × 0.025‐in SS; metal ligatures for CLA. (During the first 3 months of alignment, 0.014‐in NiTi was maintained unless replaced with the same diameter)	No (nonextraction cohort)	Conventional; no TADs or intermaxillary/auxiliary appliances during the study period	AcceleDent (bite mouthpiece; manufacturer’s instructions); force/frequency not reported in this paper 20 min/day starting the day of bonding, for the first 4 weeks	Models at t0, T1 (1 mo), T2 (2 months), T3 (3 months); IL‐1β at t0, 1 h (t1), 1 wk (t2), 3 wks (t3); pain VAS at 4 h, 24 h, 3 d, 7 d; then 4‐weekly visits	3 months (alignment phase; device used only first 4 weeks)	Primary: Alignment rate (Little’s Irregularity Index, LII); IL‐1β concentration; pain/discomfort. Secondary: GCF volume; periodontal depth (PD)	LII: study casts; Mitutoyo digital caliper (0.005 mm); intraexaminer ICC ~0.96. GCF/IL‐1β: Periotron 8000 quantification; ELISA (Invitrogen), pg/µL. Pain: 100‐mm VAS (4 h, 24 h, 3 d, 7 d)	Alignment: During Months 1–2, SLA (*p* = 0.0491 at T2) and SLA‐VA (*p* = 0.0134 at T2) were faster than CLA; no differences between SLA and SLA‐VA at any time; by month 3, groups converged. IL‐1β: SLA‐VA > SLA and CLA at t1 (*p* = 0.0209 vs. SLA; *p* = 0.0018 vs. CLA) and t2 (*p* = 0.0352 vs. SLA; *p* = 0.0029 vs. CLA); at t3 difference persisted only vs. CLA (*p* = 0.0304). Pain: No significant differences among groups at any time point. Conclusion: SL brackets align faster than CL early on; vibration increased IL‐1β but did not accelerate alignment or reduce pain
Katchooi et al. [51], USA and Canada, parallel	2 arms: (1) Active AcceleDent Aura + Invisalign (1‐week changes) (2) Sham AcceleDent + Invisalign (1‐week changes)	Randomized *N* = 27 (*A* = 13, *B* = 14 → analyzed 13/arm after 1 discontinuation); F/M = 15/12, mean age ~33.0 ± 11.8 years (groups similar)	Alignment and leveling	Invisalign; ≤25 aligners; per‐aligner movement ≤0.25 mm; 1‐week change regimen; seen every 3 weeks for fit; attachments/IPR per orthodontist; some arches “spread‐out” to match counts	No (adult aligner cohort; no specific extraction protocol)	N/A (aligners; no TADs)	Active: ~0.25 N at 30 Hz (AcceleDent Aura). Sham: identical device with zero amplitude (coupler removed) 20 min/day (company instructions); device usage downloaded each visit; mean ~115–121 min/week across groups	Every 3 weeks: check fit and dispense 3 aligners; baseline and completion iTero scans; pain NRS 1‐week diaries at start and midpoint; OHIP‐based QoL at baseline/mid/end	Through initial aligner series only (varies by case) under a 1‐week change regimen; visits q3 weeks	Primary: % completion of initial series on 1‐week regimen. Secondary: Incisor Irregularity Index (final and change), compliance, pain (NRS), OHQoL	Models: OrthoCAD on digital scans; occlusal view with 1‐mm grid; ~10x magnification; contacts measured; repeatability Dahlberg ~0.1 mm. Pain: daily NRS x7 days (start and midpoint). QoL: modified OHIP. Device and aligner compliance recorded (blue‐dot indicator; device download)	No significant differences. completion: 77% (active) vs. 85% (sham), *p* = 1.00. Alignment: final and *Δ*Irregularity Index NS between groups. Pain: only Day 3 (baseline set) differed (lower with active), others NS. QoL: NS. Conclusion: AcceleDent did not improve weekly regimen completion, final alignment, pain, or OHQoL with aligners
Leethanakul et al. [60], Thailand, SMD	2 arms: (1) Experimental side: 60 g canine distalization + vibrating electric toothbrush (2) Control side: 60 g distalization without vibration	*N* = 15 (F/M = 11/4), 19–25 years, mean 22.9 years	Canine retraction	Roth preadjusted edgewise brackets (3M gemini) 0.022" on maxillary canines/posteriors; passive SS segments to U6–U5; power arm 0.021 × 0.025 SS on canine; bilateral elastomeric chains (buccal and palatal) adjusted to net 60 g by force gauge; force reapplied at start of Month 2 and 3	Yes—bilateral maxillary first premolars (prior to retraction)	Conventional intra‐arch mechanics (no TADs)	Colgate motion electric toothbrush (rotating/vibrating head) used to deliver mechanical vibration ~125 Hz to the mesiolabial surface of the experimental canine 15 min/day total (5 min × 3 times/day) for 2 months (starting after the first retraction month)	Monthly: baseline, T0 (preretraction), T1 (1 month retraction, no vibration), T2 (1 month with vibration), T3 (2 months with vibration)	3 months of retraction (vibration during last 2 months)	Primary: IL‐1β level in GCF. Secondary: Amount/rate of canine movement (monthly and cumulative)	GCF/IL‐1β: paper‐point collection at mesial and distal sites; ELISA; concentration pg/mL. Tooth movement: palatal plug indexed to 3rd palatal ruga; displacement of canine mesial contact measured with digital caliper (0.01 mm); repeated measures; Wilcoxon tests; Dahlberg error <0.5; assessor blinded	IL‐1β (pressure sites): experimental 0.64 ± 0.33 pg/mL vs. control 0.10 ± 0.11 pg/mL, *p* < 0.001; elevations significant at T2/T3. Tooth movement (cumulative): experimental 2.85 ± 0.17 mm vs. control 1.77 ± 0.11 mm, *p* < 0.001; movement at T2 roughly doubled vs. T1 with vibration, and remained higher than control at T3. Conclusion: Vibration with light force increased IL‐1β and accelerated canine movement over 2 months
Lobre et al. [61], USA, parallel	2 arms: (1) Micropulse vibration device (AcceleDent Aura) + fixed treatment (2) Control (no device)	Randomized: 70 (35/arm). Analyzed: 58 total = 29/arm (after exclusions). F/M: not reported, age: ≥10 years (adolescents and adults), mean not reported	Alignment and leveling	Conventional fixed appliances with monthly separator/archwire adjustments; pain tracked after each adjustment. Specific bracket/wire system not detailed	No (nonextraction pain trial)	Conventional (no TADs/headgear; not part of protocol)	AcceleDent Aura: 0.25 N at 30 Hz; occlusal mouthpiece; USB port for usage logging. 20 min/day throughout the 4 months; patients marked VAS within 1 h of use. Rescue analgesics discouraged/recorded	Pain diary daily × 7 days after each monthly adjustment, then weekly for the rest of the month; compliance verified via USB	4 months (leveling/aligning phase)	Primary: VAS for overall pain and biting pain (monthly averages). Exploratory: age/sex subgroups; rescue‐medication use	VAS 0–100 mm booklets for overall and biting pain; repeated‐measures ANOVA (α = 0.05); device usage via USB; instruction to avoid analgesics except documented “rescue” doses	Significantly lower pain with vibration: overall pain *p* = 0.002, biting pain *p* = 0.003 over 4 months. Monthly means favored device in 3/4 months for biting pain and all 4 for overall pain (table/figures in paper). Pain peaked ~24 h postadjustment. Retention 83% (29/35 per arm). No harms reported
Mayama et al. [12], Japan, SMD	2 arms: (1) TM + V, static force (100 gf coil) + supplementary vibration at each monthly visit (2) TM: static force only	Analyzed *N* = 23 (F/M = 21/4), mean age 20.2±7.0 (range 13.5–40.4)	Canine retraction	Roth 0.018" brackets on canines and 2nd premolars; tubes on 1st/2nd molars; leveling with 0.014” NiTi; retraction on 0.016 × 0.022” SS withNiTi closed‐coil 100 gf from 1st molar to canine	Yes—bilateral upper first premolars	Absolute anchorage: miniscrews (Ø 1.4 mm, length 5–7 mm) between U5–U6	Prototype clinic‐applied vibrator (controller + rotary motor + bracket‐slot attachment): 5.2 ± 0.5 gf at 102.2 ± 2.6 Hz 3 min / monthly visit (in‐clinic; not a home‐use daily device)	Monthly; models each visit; up to 8 visits	Mean course 6.9 ± 2.9 months (min 1.6, max 9.9)	Primary: Canine movement/visit (3D) and estimated visits to close space. Secondary: Pain/discomfort (VAS), root resorption (crown–root ratio), adverse events	3D models (STL) from alginate casts; superimposition on palatal stable region + U7 occlusal surfaces; movement decomposed into *x*/*y*/*z* axes; LME model for longitudinal data. Pain: VAS at multiple timepoints (1 h → 14 d). Root resorption: panoramic crown–root ratio. Reliability: ICC 0.97 (movement), 0.99 (CRR)	Movement/visit: 1.21 ± 0.60 mm (TM + V) vs. 0.89 ± 0.55 mm (TM), *p* < 0.01; greater on *x* and *z* axes (most visits). Estimated visits: 4.61 ± 2.15 (TM + V) vs. 6.38 ± 3.10 (TM), *p* < 0.01 (~1.77 fewer). Pain/discomfort: no differences; VAS peaked 6–24 h then normalized by Day 7. Root resorption: no difference in crown–root ratio. Harms: none; miniscrews/brackets stable
Miles et al. [62], Australia/USA, parallel	2 arms: (1) Vibration (tooth masseuse) + fixed (2) Control (fixed only)	*N* = 66 randomized; LII cohort 33/arm; pain cohort ~31 vs. 29; age: 11.1–15.7 years, means ~13.0–13.1 years (groups similar)	Alignment and leveling	Fixed MBT 0.018″ (3M Victory). 0.014″ thermal NiTi (M5 Heaters) kept 10 weeks; retie at 5 weeks; no other adjustments; clinician blinded	No (nonextraction lower arch inclusion)	Conventional; no TADs	Tooth masseuse: 111 Hz, 0.06 N (~6.1 g); first use immediately after initial wire placement. 20 min/day minimum (patient‐directed use)	Models at start, 5 w, 8 w, 10 w; Pain VAS at: immediate, 6–8 h, 1 d, 3 d, and 7 d	10 weeks (initial alignment phase)	Primary: Little’s Irregularity Index (LII) change (rate/amount). Secondary: Pain (VAS 0–100 mm)	LII: mandibular six anterior casts at four time points; single examiner; blinded coding. Pain: five‐time point VAS; measured by blinded staff; standard stats (paired *t*‐tests; *α* = 0.05)	No significant differences at any time in irregularity or pain between groups. LII reduction at 10 w: 65% (vibration) vs. 69% (control). Conclusion: No clinical advantage of 20‐min/day vibration for early crowding resolution or pain relief
Miles and Fisher [16], Australia, parallel	2 arms: (1) AcceleDent Aura + fixed (2) Fixed only (no device)	*N* = 40 randomized (20/arm); F/M overall = 26/14 (AcceleDent 14/6, control 12/8), age range 10.5–15.6 years, mean overall 12.8 ± 1.3 years (groups 12.7 vs. 13.0)	Alignment and leveling	Fixed MBT 0.018″ (3M Victory). Initial wire 0.014″ thermal NiTi kept 10 weeks; retie at 5 weeks; no other adjustments during study window; clinician blinded	No mandibular extractions (study arch). Maxillary premolar extractions performed as part of overall treatment (outside outcome arch)	Conventional (no TADs)	AcceleDent Aura (OrthoAccel). Background spec referenced in‐text: ~30 Hz, 0.2 N (~20 g) micropulses via occlusal mouthpiece ≥20 min/day; device logs usage (median compliance 79%, mean 73.8% ± 21.7%)	Models at start, 5 w, 8 w, and 10 w; Pain VAS at baseline, 6–8 h, 24 h, 3 d, and 7 d; all charts returned; no missing data	10 weeks (initial alignment phase)	Primary: Change in mandibular anterior arch perimeter (mm). Secondary: Change in Little’s Irregularity Index (LII); discomfort (VAS); analgesic use; subgroup of “good compliers” (≥75% usage)	Arch perimeter: distal of canines → labiolingual centers canine‐to‐canine (digital caliper); LII: 5 contacts canine‐to‐canine; VAS: 0–100 mm at 5 time points; reliability excellent (ICC 0.98–1.00). Stats: median regression; analgesics by chi‐square	No significant differences at any time point for arch perimeter or LII (baseline, 5 w, 8 w, and 10 w, all NS). Discomfort: no differences at any time point. Analgesics: fewer users at 24 h with AcceleDent (*p* < 0.01); other times NS. Compliance subgroup (≥ 75%): still no differences in perimeter or LII
Pescheret [63], USA, parallel	2 arms: (1) Invisalign + AcceleDent Aura (experimental) (2) Invisalign only (control)	Analyzed *N* = 36 → 18 AcceleDent (F/M 12/6), 18 Control (F/M 10/8), age (year, mean): control ~19.2, AcceleDent ~25.5, overall ~22.4	Alignment and leveling	Invisalign; aligner change protocol: 7‐day with AcceleDent (or 10‐day if non‐compliant), 14‐day without device; routine IPR/elastics/refinements as needed; iTero scans for records	No (extraction cases excluded by eligibility)	N/A (aligner therapy; no TADs)	AcceleDent Aura: 0.25 N at 30 Hz micropulses via occlusal mouthpiece 20 min/day (extra “getting‐started” doses: 2 × 20 min on Days 0 and 1; then 20 min nightly)	Scans at T1 (baseline), T2 (~3 months), T3 (~6 months), T4 (12 months or end); pain surveys day of new aligner, daily × 3, then weekly; device usage downloaded (FastTrac)	Up to 12 months observation (end of treatment or 12 months, whichever comes first)	Primary: Little’s Irregularity Index (LII) change (upper/lower). Co‐primary: Pain (0–10 VAS). Secondary: Total treatment time, device compliance, harms	LII: OrthoCAD measurements on iTero scans (standardized magnification; excellent ICC >0.90). Pain: SurveyMonkey 0–10 VAS at specified timepoints; averaged per aligner and by periods (0–3, 3–6, and 6–12 months). Stats: normality (Shapiro–Wilk); independent/paired*t*‐tests (*α* = 0.05)	Alignment (LII): No significant differences between groups at any time point or interval differences (T1→T2, T1→T3, T1→T4). Pain: Significantly lower mean pain in AcceleDent during 6–12 months (~0.51 vs. 1.49; *p* = 0.047); no differences at 0–3 or 3–6 months. Total treatment time: no significant between‐group difference. Compliance/harms: usage logged; no adverse events reported
Reiss et al. [14], USA, parallel	2 arms: (1) AcceleDent (vibration) + fixed appliances (2) Fixed appliances only (control)	*N* = 40 randomized ITT: 20 vibration, 20 control (gender‐balanced 10/10 each), age range 15–35 years, mean at T0:21.1 years (vibration) vs. 19.7 years (control); females 22.0 years, males 18.7 years	Alignment and leveling	Lower arch: Carriere passive self‐ligating brackets 0.022 × 0.028 from LR5–LL5; molar tubes. Wires: 0.014″ Cu‐NiTi at T0 (re‐engaged at T1) → 0.014 × 0.025″ Cu‐NiTi at T2; adjustments every 4–6 weeks	No (nonextraction during study window per eligibility)	Conventional fixed‐appliance mechanics (no TADs)	AcceleDent intraoral vibrational device (occlusal mouthpiece); manufacturer‐specified micropulses (specs not numerically detailed in paper). 20 min/day instructed; usage downloaded from device. Mean compliance ≈ 53% (≈ 10.6 min/day) over T0–T3 (range 0%–104%; females ~66%, males ~41%)	T0, T1 (4–6 w), T2 (10–12 w), T3 (15–18 w); same‐time‐of‐day scheduling; bracket issues rebonded within 7 days	~15–18 weeks (~3–4 months)	Primary: Change in salivary biomarkers (17‐analyte multiplex: OPN, RANKL, SOST, OPG, MMP‐1/8/9/13, TNF‐α, IL‐1β/3/6/8/11/18, DKK1, TGF‐β1). Secondary: RMAA (mandibular anterior) and compliance	Saliva: unstimulated whole saliva, processed and assayed via Luminex multiplex; total protein normalized. RMAA: Little’s Irregularity Index on mandibular casts with digital caliper; duplicate readings, ICC >0.85. Stats: ITT; Mann–Whitney/Wilcoxon + linear mixed‐effects models; assessor blinded	No significant differences between groups for any biomarker or RMAA at T1, T2, T3; no correlation between changes in irregularity and biomarker levels; no association between RMAA and device compliance. trend: MMP‐9 and IL‐11 rose over time in control but were flat with AcceleDent (did not reach significance in ITT). No harms reported. conclusion: Supplemental vibration did not affect biomarkers or alignment rate
Siriphan et al. [44], Thailand, parallel	3 arms: (1) 30 Hz vibration + distalization (2) 60 Hz vibration + distalization (3) Distalization only (control)	*N* = 60 (groups 20/20/20). Sex overall = 13 M/47 F (per‐group M: F 3:17, 5:15, 5:15), age 18–25 years, group means ~21.6 ± 2.0, 22.1 ± 2.5, 20.9 ± 1.7	Canine retraction	Roth 0.022″ brackets; leveling/alignment then 0.016 × 0.022″ SS working archwire;NiTi closed‐coil 60 cN from 1st molar to canine; coil reactivated every 4 weeks; one canine per subject randomized for measurements	Yes—bilateral maxillary first premolars (2 weeks before starting distalization)	Conventional intra‐arch mechanics (no TADs)	Modified electric toothbrushes delivering 30 Hz or 60 Hz; calibrated: amplitude 3.85 µm, acceleration 9.81 m/ s^2^, magnitude ~0.1 cN 20 min/day. Days 1–7: applied in‐clinic by investigator; Days 8–90: home use with daily SMS reminders	Records at T1 (predistalization), T2 (24 h), T3 (48 h), T4 (7 d), T5 (3 months); appliance activation every 4 weeks	3 months	Primary: Rate of canine movement (mm/month); RANKL and OPG concentrations (and RANKL/OPG ratio). Secondary: Molar movement rate, canine rotation angulation, molar angulation	3D models: T1 vs. T5 superimposed in 3Shape OrthoAnalyzer on palatal rugae + fovea palatina; distances cusp‐tip–based; cephalometrics for angulations. GCF: PerioPaper/Periotron; ELISA (R&D Systems) for RANKL/OPG. Stats: Kruskal–Wallis (+ Dunn); method error <0.5 mm/0.5°; ICC 0.86–0.99	No significant differences among groups. Canine rate (median): 0.82 (30 Hz), 0.87 (60 Hz), 0.83 mm/month (control), *p* > 0.05. RANKL/OPG: no between‐group differences; only control‐compression RANKL rose vs T1 at 24 h/48 h/7 d. Molar movement, tipping, rotation: all NS. Conclusion: 30 or 60 Hz vibration did not increase distalization rate nor alter RANKL/OPG compared with force alone
Taha et al. [42], USA, parallel	2 arms: (1) AcceleDent Aura + fixed appliances (2) Fixed appliances only (no device)	*N* = 21 randomized → control 11, experimental 10, age adolescents 12–17 years, (means ~15.1 vs. 15.9 years)	Canine retraction	MBT 0.022‐in precoated brackets (3M Victory); working archwire 0.018‐in SS; NiTi closed‐coil (Dentos) delivering 180 g from 1st‐molar band hook to canine hook	Yes—unilateral or bilateral maxillary first premolars	Conventional intra‐arch mechanics (no TADs)	AcceleDent Aura (micropulse vibration 30 Hz) 20 min/day (evening use instructed)	**Monthly**: **T0** (start retraction), **T1** (4 w), **T2** (8 w), and **T3** (12 w)	**12 weeks**	**Primary:** Amount/rate of canine movement (mm and mm/month). **Secondary: Pain** (VAS 1–10 for 7 days postactivation), **device compliance**	**3D digital models** (iTero Element II) each visit; superimposition on **third palatal rugae** (3 points; 200% magnification) in **3Shape OrthoAnalyzer**; linear cusp‐tip displacement; **VAS** pain daily × 7 after each activation; **ICC** intra 0.99/inter 0.80;**two-sample *t*-tests**, *α* = 0.05	**No significant differences** in total or monthly tooth movement or pain.**Monthly rate (mm/mo):** control **1.21 ± 0.32** vs. vibration **1.12 ± 0.20** (overall NS); by visit: **T1** 1.12 vs. **1.39** (*p* = 0.058), **T2 1.47** vs. 0.93 (*p* = 0.028, favoring control), **T3** 1.01 vs. 1.05 (*p* = 0.879). **Pain:** similar trends, **NS** at all time points. **Compliance:** mean **56.3%**, declined over time; no correlation with movement
Woodhouse et al. [48], UK, parallel	3 arms: (1) Active AcceleDent + fixed appliances (2) Sham AcceleDent + fixed (3) Fixed‐only (no device)	*N* = 81 randomized: active 29, sham 25, control 27; F/M = 41/40, age <20 years, overall mean ~14.1 years (SD 1.7)	Alignment and leveling	Standardized MBT precoated brackets (3M Victory); archwires 0.014” → 0.018” NiTi during study; full engagement required; no bite planes, auxiliaries, elastics, headgear, or TADs	Yes—mandibular first premolars extracted as part of overall plan (eligibility)	Conventional fixed mechanics (no TADs)	AcceleDent removable device: ~ 30 Hz, ~ 0.2 N cyclic vibrational force via occlusal mouthpiece 20 min/day (instructions given; device timer present though unreliable for logging)	Pain diaries after T1 (0.014” NiTi) and T2 (0.018” NiTi): immediately, 4 h, 24 h, 72 h, 1 wk; alignment rate assessed T1→T2	Two 1‐week monitoring windows (post‐T1 & post‐T2) within early alignment; alignment rate calculated over T1→T2 interval	Primary: Maximum pain (VAS 0–100 mm) during early alignment. Secondary: Mean pain by time point, alignment rate (mm/day T1→T2),oral analgesic use/number	Pain: patient questionnaires (VAS) at specified time points; analgesic intake recorded. Alignment: Little’s Irregularity Index on mandibular casts T1 and T2; analyses with ANOVA/regression; assessor and statistician blinded	No significant differences among active, sham, and control in maximum pain (*p* = 0.282) or mean pain by time point; pain peaked at 4 and 24 h, declined by 72 h, insignificant at 1 wk. Analgesics: taken by 69% (T1) and 34% (T2); intervention effect independent of analgesia.alignment rate: no between‐group differences. conclusion: Supplemental vibration did not reduce pain or analgesic consumption, nor improve early alignment rate
Yilmaz et al. [64], Turkey, SMD	2 arms: (1) Vibration (Oral‐B HummingBird) (2) Control (no vibration)	*N* = 20 patients (7 M/13 F), age 15.08–18.58 years, mean 16.77 years	Experimental buccal tipping	Force group: self‐ligating SPEED tubes/brackets 0.022 × 0.026‐in on U4/U6; 0.017 × 0.025‐in TMA cantilever delivering 150 g buccal force (gauged). No‐force group: same anchorage without force application	Yes—maxillary first premolars extracted after the 12‐week experimental period	Transpalatal arch with bonded occlusion‐rising acrylic plates on U6 to prevent occlusal contacts; no TADs	Oral‐B HummingBird with modified tip; buccally directed vibration ~50 Hz to the mid‐buccal of the premolar 10 min/day for 12 weeks	Clinical monitoring during the 12 weeks; end point at extraction and micro‐CT analysis	12 weeks	Primary: Total root‐resorption crater volume (mm^3^). Secondary: Distribution by surface (B/P/M/D) and vertical third (cervical/mid/apical)	Extracted premolars scanned with micro‐CT (SkyScan‐1172); crater volumes quantified in Fiji/ImageJ (convex‐hull method); statistics: Wilcoxon (within‐group) and Mann–Whitney *U* (between‐group)	Vibration vs. control: No significant difference in either group. Force group totals: Vibration 0.476 mm^3^ vs. control 0.462 mm^3^ (*p* > 0.05). No‐force group totals: vibration 0.017 mm^3^ vs. control 0.031 mm^3^ (*p* > 0.05). Force vs. no‐force: Significantly greater resorption with force on both vibration and control sides (*p* ≤ 0.001). Conclusion: 50‐Hz mechanical vibration did not reduce root resorption; 150 g buccal force increased resorption

*Note:* Cu‐NiTi, copper–nickel–titanium (archwire alloy); IL‐1β, interleukin‐1 beta (pro‐inflammatory cytokine); IL‐8, interleukin‐8 (neutrophil‐chemoattractant chemokine); LCPA, long‐cone periapical radiograph; MBT, McLaughlin–Bennett–Trevisi prescription; NiTi, nickel–titanium (archwire alloy); NRS, numeric rating scale (0–10) for pain; SLA‐VA, self‐ligating appliance plus vibration appliance; SS, stainless steel (archwire); TADs, temporary anchorage devices (miniscrews); TMA, titanium–molybdenum alloy (archwire); UR1, upper right central incisor.

Abbreviations: 3D, three‐dimensional; AP, anteroposterior; CAT, clear aligner therapy; CBCT, cone‐beam computed tomography; CI, confidence interval; cN, centinewton; CT, computed tomography; d, days; ELISA, enzyme‐linked immunosorbent assay; g, gram; GCF, gingival crevicular fluid; gf, gram‐force; GIC, glass ionomer cement; Hz, hertz; ICC, intraclass correlation coefficient; IPR, interproximal reduction; ITT, intention‐to‐treat; LII, Little’s Irregularity Index; min, minutes; mm, millimeter; mm^3^, cubic millimeter; NS, not significant; OB, overbite; OHIP‐14, Oral Health Impact Profile‐14; OIIRR, orthodontically induced inflammatory root resorption; OPG, osteoprotegerin; OPN, osteopontin; OTM, orthodontic tooth movement; PCPDI, Proximal Contact Point Discrepancy Index; PD, probing depth; pg/mL, picograms per milliliter; PGE2, prostaglandin E2; RANKL, receptor activator of NF‐κB ligand; RCT, randomized controlled trial; RMAA, rate of mandibular anterior alignment; SD, standard deviation; SLA, self‐ligating appliance; SMD, split‐mouth design; VAS, visual analog scale; w, week; Wks, weeks.

### 3.3. Risk of Bias in the Included Studies

Risk of bias was evaluated at the outcome level across seven prespecified variables—pain and discomfort, oral health‐related quality of life measured with the OHIP‐14 instrument, analgesic use, root resorption, biomarkers, periodontal indices, and tooth mobility—using the Cochrane RoB 2 framework.

In total, 37 outcome‐level judgments were issued. Only two studies were rated as having a low risk of bias, both related to root resorption and biomarker outcomes. Most judgments (33/37; 89%) were rated as “some concerns,” with issues predominantly located in Domain 5 (selection of the reported result) in 81.08% of outcomes, Domain 4 (measurement of the outcome) in 70.27%, and Domain 1 (randomization process) in 67.56%. Two judgments were rated as “high risk of bias” for both pain and discomfort, due to deviations from the intended interventions captured in Domain 2.

**Figure 2:**
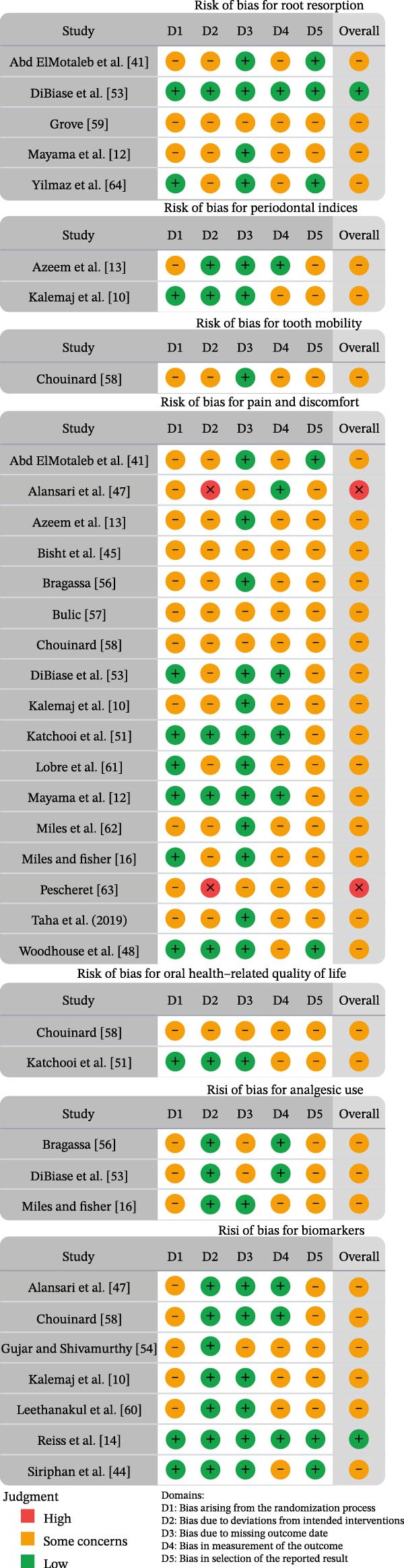
Risk of bias summary of RCTs: the review authors’ judgments about each item of the risk of bias for the included studies using the RoB 2 tool.

**Figure 3:**
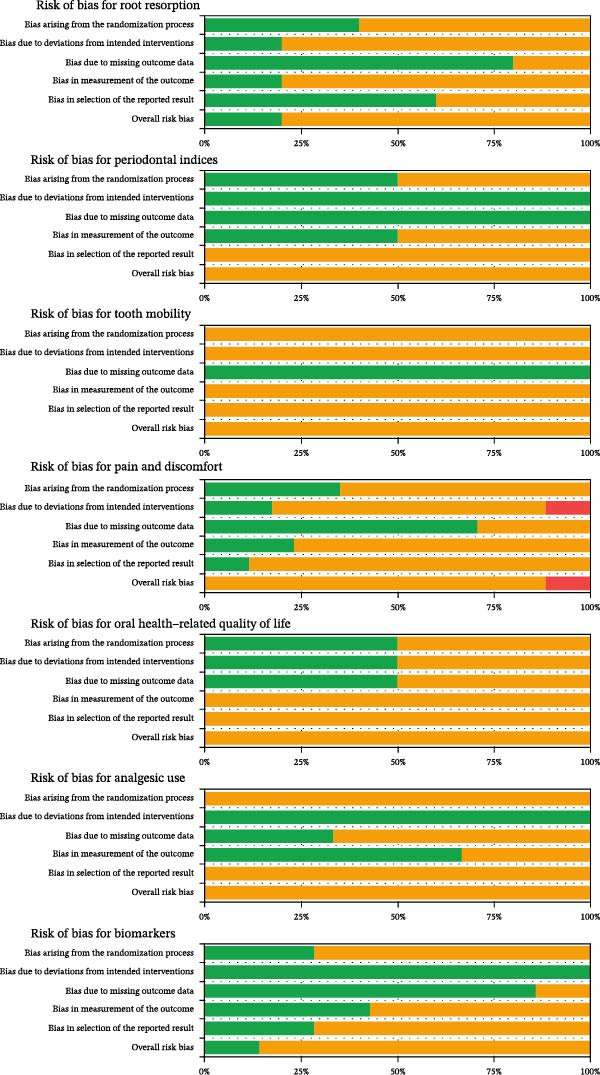
The overall risk of bias score for each field of RCTs: the review authors’ judgments about each item of the risk of bias, presented as percentages across all the studies included.

RoB 2 assessments are summarized in Figures 2and 3, which were generated using the RoB‐Var (risk of bias by variables; MRS Edition) tool [65]. Detailed justifications for each domain and outcome are provided in Supporting Information 5: Table S5.

### 3.4. Main Findings on the Safety and Adverse Effects of VA‐OTM

The principal outcomes from RCTs are organized into three domains (Tables 3 and 4): (1) clinical safety end points (root resorption, periodontal indices, and tooth mobility), (2) tolerability/acceptability outcomes, and (3) biological response/surrogate markers.

**Table 3:** Clinical safety end points, tolerability outcomes, and biological response markers of vibration‐assisted orthodontic tooth movement (pooled RCT data).

Study	Ortho goal				Study	Method of measurement	Treatment effect (device vs. control)	Number of events	*p* value
Clinical safety end points									
Root resorption	Canine retraction (2 RCTs)	Parallel RCT	Fixed appliance		Abd ElMotaleb et al. [41]	CBCT (~0.3 mm voxel); linear root/crown length (cusp–apex) mm, linear (3D source) Pre‐ vs. postretraction (~4 months)	Root resorption (change in root length, mm): device 0.8 ± 0.7 mm vs. control 0.6 ± 1.0 mm; difference 0.24 mm—not significant ⇒ no device effect	64 canines	0.289
		SMD			Mayama et al. [12]	Panoramic (2D); Crown–Root Ratio (CRR) Ratio (derived linear), index Pre‐ vs. postretraction (monthly up to ~8)	Root resorption (crown–root ratio): start 0.59 ± 0.02 (control) vs. 0.58 ± 0.02 (vibration); end 0.59 ± 0.02 on both sides—no difference between groups ⇒ no increase in resorption with vibration	46 canines	NS
	Alignment and leveling (1 RCT)	Mandible with extraction	Fixed appliance		DiBiase et al. [53]	Periapicals (2D); OIIRR at UR1 mm, linear T1 = start of alignment (baseline periapical); T3 = end of alignment at insertion of 0.019 × 0.025″ SS. Mean T1→T3 interval: 201.6 days	Root resorption (mm, maxillary right central incisor): device 1.09 ± 0.64 vs. control 1.00 ± 0.90; difference ~+0.09 mm—not significant. Severe >2 mm: 11% (device) vs. 17% (control)—not significant	~72 incisors (UR1)	NS *p* > 0.05
	Experimental buccal tipping (2 RCTs)	SMD	Fixed appliance		Grove (thesis) [59]	Micro‐CT (3D) of extracted premolars; total resorption crater volume mm^3^, volumetric After 4 weeks of 150 g buccal force	Treatment effect (device vs. control) on root resorption: mean total volume per tooth 0.261 mm^3^ with device vs. 0.389 mm^3^ control; difference −0.128 mm^3^ (~33% less with vibration)	28 premolars	0.003
					Yilmaz et al. [64]	Micro‐CT (3D); total resorption crater volume mm^3^, volumetric After 12 weeks	Root resorption (crater volume mm^3^): with force 0.476 vs 0.462 (device vs control; Δ = + 0.014 mm^3^, ns), and without force 0.017 vs 0.031 (Δ = −0.014 mm^3^, ns)—no proven vibration effect.	40 premolars	NS *p* > 0.05
Periodontal indices	Canine retraction	SMD	Fixed appliance		Azeem et al. [13]	Plaque index (PI): full‐mouth plaque scoring; digital caliper methods used for tooth movement; PI recorded per protocol PI recorded at R0 (baseline), R1 (1 month retraction), R2 (2 months), R3 (3 months)	Periodontal indices – plaque index (PI): no significant differences between vibration and control across R0–R3 (*p* > 0.05). Overall plaque was low: PI = 0 in 93% of sites, PI = 1 in 6.82%, PI = 2 in 0.18%	28	NS *p* > 0.05
	Alignment and leveling	Mandible without extraction	Fixed appliance		Kalemaj et al. [10]	Periodontal probing depth (PD) (mm); GCF volume also recorded (Periotron) Periodic checks during the 3‐month alignment phase (same clinical visits as LII/biomarker collection)	No significant differences among CLA, SLA, and SLA‐VA; periodontal probing depth (PD) remained within healthy limits at all time points, with modified sulcus bleeding index <25% throughout treatment	33	NS *p* > 0.05
Tooth mobility	Alignment and leveling	Mandible without extraction	Fixed appliance		Chouinard [58]	Periotest readings on selected teeth (incisors/canines/premolars) T0 baseline; T1 5–6 wks; T2 10–12 wks; T3 15–18 wks	Periotest: no device effect on mobility; early spike at incisors, then declines. Net change T0→T3: incisors +3.56 vs. +4.48, canines +3.62 vs. +2.97, premolars +1.09 vs. +1.42 (device vs. control)	23	NS *p* > 0.05
Tolerability/acceptability outcomes									
Pain/discomfort	Canine retraction (4 RCTs)	Parallel (2 RCTs)		Fixed appliance	Abd ElMotaleb et al. [41]	VAS 0–10 at 0, 24, 72 h, 7 d after activation	Median scores—4 vs. 5 (same day), 4 vs. 5 (24 h), 2 vs. 3 (72 h), 0 vs. 0 (7 days) no difference across all time points	32	0.721 0.882 0.378 0.964
					Taha et al. [42]	VAS 1–10 for 7 days after each activation	Slightly higher pain with the device on Day 1 and Days 3–6, converging by Day 7 overall, no significant difference vs. control	21	NS *p* > 0.05
		SMD (2 RCTs)			Azeem et al. [13]	VAS 100 mm (0–100); daily × 7 post‐activation, then weekly to 1 month	No difference between vibration and control sides; overall monthly means ~3.31 (R1), 3.49 (R2), and 3.78 (R3) with no device‐related improvement	28	NS *p* > 0.05
					Mayama et al. [12]	VAS 0–100 at 1 h, 6 h, 24 h, 3 d, 7 d, and 14 d after each monthly visit	No difference between vibration and control at any time. Peak at 6 h: 15.45 ± 14.66 (control) vs. 15.95 ± 15.01 (vibration) At 24 h: 14.32 ± 16.35 vs. 14.78 ± 16.69 Scores declined to baseline by Day 7	23	NS *p* > 0.05
	Alignment and leveling (11 RCTs)	Mandible without extraction (6 RCTs)		Fixed appliance	Bisht et al. [45]	VAS 0–10; 1 and 2 h postarchwire at T0, T1 (~6 w), T2 (~12 w)	VAS after archwire insertion: no difference at 1 h across groups; at 2 h, pain was lower with vibration at all visits: T0: vibration ≈2.17–2.33 vs. control/placebo ≈5.83–6.83. T1: vibration ≈1.50 vs. control/placebo ≈5.00–5.67. T2: vibration ≈1.00–1.17 vs. control/placebo ≈4.50–5.00	36	< 0.001
					Chouinard [58]	VAS 0–10; daily for 7 days after each visit	No significant difference between vibration and control. Weekly means (mean ± SD): T0 : 40.94 ± 28.18 (vibration) vs. 34.37 ± 14.16 (control) T1 : 16.92 ± 22.20 (vibration) vs. 14.00 ± 16.33 (control) T2 : 28.28 ± 24.62 (vibration) vs. 40.96 ± 22.25 (control)—no device benefit	23	NS *p* > 0.05
					Kalemaj et al. [10]	VAS 100 mm at 4 h, 24 h, 3 d, and 7 d	No significant differences between vibration and control at any time. Means (mean ± SD): 4 h: CLA 58.1 ± 14.5, SLA 50.7 ± 11.0, SLA‐VA 55.3 ± 14.8 24 h: CLA 41.1 ± 10.5, SLA 36.9 ± 7.9, 3SLA‐VA 9.1 ± 10.5 3 days: CLA 19.6 ± 8.8, SLA 17.6 ± 5.0, SLA‐VA 17.1 ± 4.8 7 days: CLA 10.0 ± 5.6, SLA 8.7 ± 4.7, SLA‐VA 8.6 ± 6.3 peak at 4 h, then decline to Day 7	33	NS *p* > 0.05
					Miles et al. [46]	VAS 0–100 mm at immediate, 6–8 h, 1 d, 3 d, and 7 d postarchwire	VAS/100 (control mm → device mm): immediately 8.1→12.4; 6–8 h 39.6→40.4; 1 day 47.6→41.5; 3 days 19.9→18.8; 7 days 5.5→4.0. No differences at any time	58 analyzed	NS *p* > 0.05
					Miles and Fisher [16]	VAS 0–100 mm at baseline, 6–8 h, 24 h, 3 d, and 7 d	No significant difference. median values (device mm vs. control mm): baseline 8.0 vs. 9.5; 6–8 h 46.3 vs. 46.0; 24 h 50.5 vs. 54.0; 3 days 21.5 vs. 22.1; 7 days 2.6 vs. 2.1. No differences at any time	40	NS *p* > 0.05
				Aligner	Alansari et al. [47]	NRS 0–10; Days 1 and 3 after each aligner change	Lower pain with 7‐day vibration on Day 1, and lower than 7‐sham on Day 3; other comparisons are similar. Day 1: control 4.19 ± 0.71; 7‐sham 4.60 ± 1.13; 7‐vibration 3.39 ± 1.35; 5‐vibration 3.70 ± 0.95. Day 3: control 2.42 ± 0.64; 7‐sham 2.98 ± 1.18; 7‐vibration 1.96 ± 0.90; 5‐vibration 2.21 ± 0.91	54 (completers)	<0.05 (varies by point)
		Mandible with extraction (1 RCT)		Fixed appliance	Woodhouse et al. [48]	VAS 0–100 mm at immediate, 4 h, 24 h, 72 h, 1 wk after T1 and T2	No differences (AcceleDent vs. sham vs. control) Maximum pain (mean ± SD): overall 72.96 ± 21.59 mm; by group: 76.28 ± 18.86 (vibration), 67.32 ± 23.81 (sham), 74.63 ± 21.95 (control). Time course: peaks at 4–24 h, declines by 72 h, and is minimal by 7 days	81	*p* = 0.282 (max pain)
		Both jaws without extraction (4 RCT)		Fixed appliance	Bulic (Thesis) [57]	VAS 0–10/FACES; Day 0, Days 1–3 postadjustment, then weekly until next visit	NRS/VAS 0–10 (mean of days 1–3 post‐visit): T1 (0–3 months): control 3.69 vs. device 3.69; T2 (3–6 mo): control 2.95 vs. device 1.36; T3 (6–12 months): control 1.70 vs device 0.66 lower with the device after month 3 (at T2 and T3)	61	*p* = 0.006 (at T2 and T3)
				Aligner	Bragassa [56]	Faces/NRS at study visits: T4‐days, T2‐weeks, T6‐weeks, T12‐weeks	Pain/discomfort faces pain scale 0–10: mean scores were low (< 2.5/10) and declined over time; no analgesic effect of vibration overall. The only difference occurred at T2‐weeks, with G2 (4‐day, no vibration) reporting higher pain than G1 (14‐day)	33	NS *p* > 0.05
					Katchooi et al. [51]	NRS 0–10; daily × 7 after the baseline week, and repeated daily × 7 at mid‐series	No overall significant differences; the only exception was Day 3 (baseline week) with lower pain in the active device: 2.3 ± 1.2 vs. 4.2 ± 2.4. Reference points: Day 1: 3.4 ± 2.4 vs. 4.7 ± 3.1 (ns), and weekly pain sum baseline 15.3 ± 9.2 vs 21.6 ± 14.1 (ns)	26	NS *p* > 0.05
					Pescheret [52]	VAS 0–10/ FACES; day of new aligner, daily × 3, then weekly (up to 12 months)	No significant difference up to 6 months lower with the device at 6–12 months. Means: 0–3 mo: control 1.02 vs. device 0.91; 3–6 months: 0.99 vs. 0.61; 6–12 months: 1.49 vs. 0.51 (clearer reduction with device)	36	0.047 (6–12 months)
		Unspecified target			Lobre et al. [61]	VAS 0–100 mm; daily × 7 after each monthly adjustment, then weekly for the rest of the month (over 4 months)	VAS/100 mm—lower with vibration: overall pain (device → control) Month 1: 8.78→17.20, Month 2: 4.62→13.11, Month 3: 3.83→9.22, Month 4: 2.54→8.80 Biting pain lower from Month 2 onward	58	Overall *p* = 0.002 Biting *p* = 0.003
Oral health–related quality of life (OHIP‐14)	Alignment and leveling (2 RCTs)	Mandible without extraction		Fixed appliance	Chouinard [58]	OHIP‐14 at baseline, mid‐treatment, and end (~3–4 months)	OHIP‐14 (lower is better): device lower than control at all visits but not significant—T0 : 5.24 ± 4.96 vs. 7.33 ± 4.06; T1 : 5.50 ± 3.27 vs. 7.27 ± 3.26; T2 : 4.40 ± 4.19 vs. 5.52 ± 3.30; T3 : 3.18 ± 2.79 vs. 4.71 ± 2.88 (all nonsignificant)	23	NS *p* > 0.05
		Both jaws without extraction		Aligner	Katchooi et al. [51]	Modified OHIP at baseline, midpoint, and end of initial series	OHIP‐14 (lower is better): no significant differences between device and control at any visit; item means ~1.0–2.0/5. Example (uncomfortable eating): baseline 1.6 vs. 1.5, midpoint 1.9 vs. 1.9, end 1.7 vs. 1.8; the only exception was “diet unsatisfactory” at baseline 1.1 vs. 1.6, favoring the device (baseline difference only)	26	NS *p* > 0.05
Analgesic use	Alignment and leveling (3 RCTs)	Mandible without extraction		Fixed appliance	Miles and Fisher [16]	Analgesic reporting with pain VAS at baseline, 6–8 h, 24 h, 3 d, 7 d; chi‐square for group comparisons	Only difference at 24 h favoring device—60% vs. 95%; other time points not different: baseline 35% vs. 35%, 6 h 70% vs. 75%, 3 days 15% vs. 20%, 7 days 0% vs. 5%	40	*p* < 0.01(24 h) *p* > 0.05 other time points
		Mandible with extraction		Fixed appliance	Woodhouse et al. [48]	Pain diaries after T1 and T2 (immediate, 4 h, 24 h, 72 h, 1 wk), including analgesic intake and counts	No significant differences among device, sham, and control during the week after T1 and T2 (intervention × analgesia interaction *p* = 0.883; all secondary comparisons *p* > 0.003). Overall analgesic use was 55/80 (~69%) at T1 and 26/77 (~34%) at T2	81	NS
		Both jaws without extraction		Aligner	Bragassa [56]	Analgesic intake captured alongside Faces/NRS pain questionnaires across 12 weeks	No significant differences among groups overtime (*p* = 0.612). Peak at T1: G1 = 14%, G2 = 17%, and G3 = 27%; lower thereafter	33	NS *p* = 0.612
Biological response/surrogate markers									
Biomarkers	Alignment and leveling (4 RCTs)	Mandible without extraction	Fixed appliance		Chouinard [58]	Saliva, ELISA (e.g., IL‐1β, IL‐8; TNF‐α often undetectable) Baseline, mid / end of ~3–4 months	Vibration showed no meaningful difference in salivary biomarkers vs. control; for example, at T3, IL‐1β was 26.98 vs. 132.73 pg/mL, and IL‐8 was 299.11 vs. 384.32 pg/mL; TNF‐α was below the detection limit in all samples	23	NS *p* > 0.05
					Kalemaj et al. [10]	GCF, IL‐1β conc. and GCF volume (ELISA / Periotron) t0 (baseline), t1 (1 h), t2 (1 wk), t3 (3 wks)	Biomarkers—IL‐1β (pg/µL): at 1 h higher with vibration 48.60 vs. 42.94 (SLA) and 41.12 (CLA); at 1 week, 41.42 vs. 35.80 and 33.67 (higher with device); at 3 weeks, 33.33 vs. 30.11 and 28.82 (higher than CLA only); no baseline differences	33	*p* = 0.02–0.001 (time point‐specific)
					Reiss et al. [14]	Saliva, 17‐plex (OPN, RANKL/OPG, SOST, MMP‐s, IL‐s, TNF‐α, DKK1, TGF‐β1), flow, pH (Luminex), T0, 3 w, 6 w	Biomarkers—IL‐1β (pg/mL): transient rise with vibration at 2 weeks (28.4 ± 10.3 vs. 22.9 ± 8.7, *p* > 0.05), returning to similar levels by 4 months (19.6 ± 7.4 vs. 20.1 ± 7.9, *p* > 0.05). No significant difference at any time point	40	NS *p* > 0.05
			Aligner		Alansari et al. [47]	GCF, multiplex cytokines/bone‐remodeling markers (ELISA / Luminex) At the end of the 2nd aligner under 14‐day vs. 7‐day (sham/vibration) protocols	Biomarkers (GCF): daily vibration (5‐ and 7‐day groups) produced significant increases (*p* < 0.05) in cytokines/chemokines/bone‐remodeling factors vs baseline, control, and 7‐sham No difference between 5‐ and 7‐vibration	54	<0.05 at reported points
	En masse retraction (maxillary anterior) (1 RCT)	Parallel	Fixed appliance		Gujar and Shivamurthy [54]	GCF, PGE2 (ELISA) T0, 30, 60, 90 days	Biomarker—PGE_2_ (pg/mL, GCF): consistently highest with 150 Hz vs. control, with significant group differences. Examples: T1 (30 days) 982.8 (150 Hz) vs. 763.1 (125 Hz) vs. 421.5 (control); T2 (60 days) 862.6 vs. 750.8 vs. 371.1; T3 (90 days) 805.6 vs. 531.8 vs. 355.7—marked increase with 150 Hz	30	≤ 0.03 (pairwise at several time points)
	Canine retraction (2 RCTs)	SMD	Fixed appliance		Leethanakul et al. [60]	GCF, IL‐1β (ELISA) at compression/tension sites Monthly: T0 (pre‐ret.), T1 (1 mo no vib.), T2 (1 mo vib.), T3 (2 mo vib.)	Biomarker—IL‐1β (pg/mL, GCF): higher with vibration; at pressure sites, the mean was 0.64 ± 0.33 vs 0.10 ± 0.11 in controls (*p* < 0.001), with significant rises also at T2 and T3	15	< 0.001
		Parallel			Siriphan et al. [44]	GCF, RANKL, OPG (ELISA) T1 (pre‐dist.), 24 h, 48 h, 7 d, 3 months	Biomarkers (GCF—RANKL/OPG): no significant differences among 30 Hz, 60 Hz, and control for RANKL, OPG, or RANKL/OPG ratio at any time point. Note within control: at the compression side, RANKL rose transiently from ~189 (T1) to ~450 pg/mL at 24 h, remaining higher at 48 h and 7 d (*p* < 0.001)—no added effect from vibration	60	NS *p* > 0.05

*Note:* Luminex, multiplex immunoassay platform; NRS, numeric rating scale (0–10) for pain; periotest, tooth mobility measurement device; R1–R3, review/recall visits 1–3; SLA‐VA, self‐ligating appliance plus vibration appliance; UR1, upper right central incisor.

Abbreviations: CBCT, cone‐beam computed tomography; CI, confidence interval; CLA, conventional ligation appliance; CRR, crown–root ratio; d, days; ELISA, enzyme‐linked immunosorbent assay; GCF, gingival crevicular fluid; IL‐1β, interleukin‐1 beta; IL‐8, interleukin‐8; LII, Little’s Irregularity Index; mm, millimeter; mm^3^, cubic millimeter; NS, not significant; OIIRR, orthodontically induced inflammatory root resorption; OPG, osteoprotegerin; PD, probing depth; Pg/mL, picograms per milliliter; PGE2, prostaglandin E2; RANKL, receptor activator of NF‐κB ligand; RCT, randomized controlled trial; SD, standard deviation; SLA, self‐ligating appliance; SMD, split‐mouth design; VAS, visual analog scale.

**Table 4:** Summary of findings table according to the GRADE guidelines for the included RCTs.

Outcome domain		Number of studies	Risk of bias	Inconsistency	Indirectness	Imprecision	Number of patients	Summary of effect	Overall certainty
Clinical safety end points									
Root resorption metrics	Canine retraction	2 RCTs	Serious (−1)	Not serious (0)	Not serious (0)	Serious (−1)	110	2 RCTs found no significant differences among groups	⨁⨁◯◯^a^ Low
	Leveling and alignment	1 RCT	Not serious (0)	Not serious (0)	Not serious (0)	Serious (−1)	72	1 RCT found no significant differences among groups	⊕⊕⊕◯^b^ Moderate
	Experimental buccal tipping	2 RCTs	Serious (−1)	Serious (−1)	Not serious (0)	Serious (−1)	34	1 RCT found vibration reduces total resorption crater volume 1 RCT found vibration did not reduce total resorption crater volume	⨁◯◯◯^c^ Very low
Periodontal indices (PI/GI/BOP)	Canine retraction	1 RCT	Serious (−1)	Not serious (0)	Not serious (0)	Serious (−1)	28	No significant difference in PI between vibration and nonvibration sides at any visit	⨁⨁◯◯^d^ Low
	Leveling and alignment	1 RCT	Serious (−1)	Not serious (0)	Not serious (0)	Serious (−1)	33	No significant differences reported in PD among groups; GCF volume also NS	⨁⨁◯◯^e^ Low
Tooth mobility	Leveling and alignment	1 RCT	Serious (−1)	Not serious (0)	Not serious (0)	Serious (−1)	23	Daily vibration did not alter tooth mobility compared with the control during early alignment.	⨁⨁◯◯^f^ Low
Tolerability/acceptability outcomes									
Pain and discomfort (VAS/NRS)	Canine retraction	4 RCTs	Serious (−1)	Not serious (0)	Not serious (0)	Serious (−1)	104	No important difference in pain on the same day/24 h/72 h/7 days or over 12 weeks; VAS differences near zero and nonsignificant	⨁⨁◯◯^g^ Low
	Leveling and alignment	12 RCTs	Very Serious (−2)	Serious (−1)	Not serious (0)	Serious (−1)	539	7 RCTs found no significant differences among groups 5 RCTs found lower pain with vibration	⨁◯◯◯^h^ Very low
Oral health–related quality of life (OHIP‐14)	Leveling and alignment	2 RCTs	Serious (−1)	Not serious (0)	Not serious (0)	Serious (−1)	49	Daily vibration did not improve oral health QoL vs. control	⨁⨁◯◯^i^ Low
Analgesic use	Leveling and alignment	3 RCTs	Serious (−1)	Serious (−1)	Not serious (0)	Serious (−1)	154	2 RCTs found no significant differences among groups 1 RCTs found analgesic use at 24 h was lower with vibration	⨁◯◯◯^j^ Very low
Biological response/surrogate markers									
Biomarkers (GCF panels; RANKL/OPG)	Canine retraction	2 RCTs	Serious (−1)	Serious (−1)	Not serious (0)	Serious (−1)	75	1 RCT found that adding vibration to light force raised IL‐1β and accelerated retraction. 1 RCT found no differences between groups	⨁◯◯◯^k^ Very low
	Leveling and alignment	4 RCTs	Serious (−1)	Serious (−1)	Not serious (0)	Serious (−1)	150	2 RCTs found vibration associated with elevated GCF biomarkers 2 RCTs found no between‐group differences	⨁◯◯◯^l^ Very low
	En masse retraction	1 RCT	Serious (−1)	Not serious (0)	Not serious (0)	Serious (−1)	30	1 RCT found vibration associated with elevated GCF biomarkers	⨁⨁◯◯^m^ Low


Abbreviations: BOP, bleeding on probing; CAT, clear aligner treatment; GCF, gingival crevicular fluid; GI, gingival index; GRADE, Grading of Recommendations, Assessment, Development, and Evaluation; OPG, osteoprotegerin; PAIs, physical adjunctive interventions; PI, plaque index; PROs, patient‐reported outcomes; QoL, quality of life; RANKL, receptor activator of nuclear factor κB ligand; RCTs, randomized controlled trials.

^a^Decline one level for risk of bias (some concern in the randomization process [12, 41], deviations from interventions [12, 41], measurement of outcomes [12, 41], selection of the reported result [12]), and one level for imprecision^p^.

^b^Decline and one level for imprecision^p^.

^c^Decline one level for risk of bias (some concern in the randomization process [59], deviations from interventions [59, 64], missing outcome data [59], measurement of outcomes [59, 64], selection of the reported result [59]), one level for Inconsistency^n^, and one level for imprecision^p^.

^d^Decline one level for risk of bias [13] (some concern in the randomization process and selection of the reported result), and one level for imprecision^p^.

^e^Decline one level for risk of bias [10] (some concern in the measurement of outcomes, and selection of the reported result), and one level for imprecision^p^.

^f^Decline one level for risk of bias [58] (some concern in the randomization process, deviations from interventions, measurement of outcomes, and selection of the reported result), and one level for imprecision^p^.

^g^Decline one level for risk of bias (some concern in the randomization process [13, 41, 42], deviations from interventions [13, 41, 42], measurement of outcomes [13, 41, 42], selection of the reported result [12, 13, 42]), and one level for imprecision^p^.

^h^Decline two levels for risk of bias (some concern in the randomization process [10, 45–58, 58, 59, 62–66], deviations from interventions [10, 16, 45, 56–58, 61, 62], missing outcome data [45, 47, 57, 58, 63], measurement of outcomes [10, 16, 45, 48, 56–58, 61–63], selection of the reported result [10, 16, 45, 47, 51, 56–58, 61–63], with high risk in deviations from interventions [47, 63]), one level for inconsistency^n^, and one level for imprecision^p^.

^i^Decline one level for risk of bias (some concern in the randomization process [58], deviations from interventions [58], missing outcome data [58], measurement of outcomes [51, 58], selection of the reported result [51, 58]), and one level for imprecision^p^.

^J^Decline one level for risk of bias (some concern in the randomization process [16, 48, 56], missing outcome data [16, 48], measurement of outcomes [56], selection of the reported result [16, 48, 56]), one level for Inconsistency^n^, and one level for imprecision^p^.

^k^Decline one level for risk of bias (some concern in the randomization process [60], measurement of outcomes [44, 60], selection of the reported result [60]), one level for Inconsistency^n^, and one level for imprecision^p^.

^l^Decline one level for risk of bias (some concern in the randomization process [10, 47, 58], measurement of outcomes [10], selection of the reported result [10, 47, 58]), one level for Inconsistency^n^, and one level for imprecision^p^.

^m^Decline one level for risk of bias [54] (some concern in the randomization process, missing outcome data, measurement of outcomes, and selection of the reported result), and one level for imprecision^p^.

^n^Wide variance of point estimates across studies.

^o^Indirectness: short assessment duration (study time frame), and a difference between desired (patient’ important) and measured outcomes.

^p^Imprecision: limited number of trials and sample size.

#### 3.4.1. Clinical Safety End Points

##### 3.4.1.1. Root Resorption

Across settings, adjunctive vibration did not demonstrate a consistent increase in root resorption compared with controls (Table 3). In canine retraction (GRADE: low), mean RR values were comparable between groups with no statistically significant difference [41], and the crown–root ratio (CRR) remained unchanged over follow‐up (Table 3) [12]. During leveling and alignment (GRADE: moderate), mean differences were small and the frequency of severe RR (>2 mm) did not differ significantly between groups (Table 3) [53]. In experimental buccal tipping (GRADE: very low), findings were heterogeneous: one micro‐CT study reported smaller resorption crater volume with vibration [11], whereas another trial reported no significant differences with or without force application (Table 3) [55].

##### 3.4.1.2. Periodontal Indices (GRADE: Low)

Periodontal indices remained stable and clinically unremarkable across trials, with no meaningful between‐group differences reported (Table 3). For example, plaque index showed no significant separation between groups in one trial [13], and PD and BOP remained within clinically healthy limits throughout follow‐up in another [10] (Table 3).

##### 3.4.1.3. Tooth Mobility (GRADE: Low)

Periotest—scale: PTVs; teeth assessed: incisors, canines, premolars at T0, T1 = 5–6 weeks, T2 = 10–12 weeks, and T3 = 15–18 weeks.

Tooth mobility assessed using periotest showed no significant effect of vibration over short‐ to mid‐term follow‐up, with both groups demonstrating similar time‐related changes [15] (Table 3).

#### 3.4.2. Tolerability/Acceptability Outcomes

##### 3.4.2.1. Pain and Discomfort

PROs—scales: VAS 0–100 mm, NRS 0–10, or faces scale 0–10.

Across 16 RCTs, vibration did not consistently reduce orthodontic pain (Table 3). In canine retraction, trials generally reported no statistically significant between‐group differences at early or later time points (GRADE: low) [12, 13, 41, 42]. During leveling and alignment with fixed appliances, results were mixed: one RCT reported lower early pain scores with vibration at very early assessments [45], whereas several others found no significant differences from baseline through 7 days (GRADE: very low) [10, 15, 16, 46]. In mandibular extraction cases treated with fixed appliances, one RCT reported no difference in maximum pain (GRADE: very low) [48]. A monthly‐assessed trial reported lower pain scores across months 1–4 with vibration (GRADE: very low) [9].

With clear aligners (four RCTs; GRADE: very low), two trials reported small early advantages at Days 1–3 [47, 51], whereas two studies reported no early differences; one trial reported a reduction only at longer follow‐up (Table 3) [50, 52]. Overall, pain peaked within 4–24 h after activation and declined toward baseline by 72 h to 7 days; across appliance types, evidence for a clinically robust analgesic effect was inconsistent (Table 3).

##### 3.4.2.2. Oral Health–Related Quality of Life (OHIP‐14; GRADE: Low)

Two trials assessing OHRQoL (OHIP‐14 or modified OHIP) showed numerical improvement over time in both groups, with no statistically significant between‐group differences at assessed time points [15, 51] (Table 3).

##### 3.4.2.3. Analgesic Use (GRADE: Very Low)

Analgesic consumption was reported in limited trials. One RCT found a lower proportion of participants taking analgesics within the first 24 h in the vibration group, while no consistent between‐group differences were observed at later time points [16] (Table 3).

#### 3.4.3. Biological Response/Surrogate Markers

##### 3.4.3.1. Biomarkers

Across seven RCTs, biomarker findings were heterogeneous across matrices (saliva vs. gingival crevicular fluid), clinical stages, and vibration protocols (Table 3). Some salivary studies during alignment reported no significant between‐group differences in biomarkers, including IL‐1β (GRADE: very low) [14, 15]. Conversely, individual trials reported statistically significant increases in IL‐1β in GCF with vibration at early sampling points (GRADE: very low) [10], and aligner‐based studies reported significant changes in multiple cytokines compared with control/sham (GRADE: very low) [47]. During en masse retraction, one trial reported consistent increases in PGE_2_ with higher‐frequency vibration (GRADE: low) [54]. In canine retraction, one study reported elevated IL‐1β with vibration [43], and another reported no significant RANKL/OPG differences (GRADE: very low) [43]. Detailed biomarker estimates by marker and time point are provided in Table 3.

## 4. Discussion

This systematic review provides a focused appraisal of the safety profile and adverse effects of VA‐OTM, based exclusively on RCTs with outcome‐level risk‐of‐bias assessment. Overall, the evidence is cautiously reassuring within the constraints of predominantly low to very low certainty data and short‐ to medium‐term follow‐up: vibration does not appear to introduce clinically meaningful harm with respect to pain, oral health–related quality of life, root resorption, periodontal status, or tooth mobility, and any observed between‐group differences are generally small and protocol‐dependent. Favorable effects on patient‐centered outcomes are modest and inconsistent, and variability in appliance type, force systems, and vibration parameters often exerts a stronger influence than the adjunctive vibration itself. The discussion below synthesizes these patterns across prespecified outcomes, situates them within the broader literature on adjunctive acceleration methods, and outlines the principal clinical implications and research priorities arising from this safety‐focused evidence base.

### 4.1. Clinical Safety End Points

#### 4.1.1. Root Resorption

Across Abd ElMotaleb et al. [41], Mayama et al. [12], and DiBiase et al. [53], vibration did not produce a clinically relevant increase in root resorption. Between‐group differences were small, statistically nonsignificant, and within the range expected for conventional orthodontic treatment. This is consistent with the understanding that root resorption is driven chiefly by force magnitude, duration of loading, and individual susceptibility, whereas the clinical vibration dose is likely below the threshold needed to substantially alter cementum or bone remodeling.

Experimental micro‐CT studies (e.g., Grove [11] and Yilmaz et al. [55]) have reported subtle changes in crater volume under highly controlled conditions, but these differences do not translate into clear long‐term clinical effects. Overall, vibration appears to be root‐resorption neutral under the studied conditions: it does not exacerbate resorption, and current evidence does not substantiate a consistent protective effect. However, longer‐term data are still needed to exclude rare or cumulative effects on root integrity.

#### 4.1.2. Periodontal Indices

Across Azeem et al. [13] and Kalemaj et al. [10], adjunctive vibration did not compromise periodontal clinical parameters. Plaque index remained low and PD and BOP stayed within healthy ranges, with no meaningful differences between vibration and control groups. This likely reflects that periodontal status is driven primarily by baseline periodontal health, oral‐hygiene practices, and stringent eligibility criteria, whereas the clinical vibration dose is low in magnitude and brief in duration, making additional tissue trauma or impaired plaque control unlikely. Collectively, these findings support the periodontal safety of vibration within the protocols studied, while acknowledging that all data are short‐ to medium‐term.

#### 4.1.3. Tooth Mobility

In the trial by Chouinard [15], adjunctive vibration did not meaningfully affect tooth mobility. PTVs for incisors, canines, and premolars increased slightly over time in both the vibration and control groups and remained statistically comparable, indicating preserved periodontal support without device‐related loosening. Given that periotest measurements primarily reflect PDL function and alveolar bone integrity—and in the absence of concomitant increases in root resorption or periodontal deterioration—vibration appears mobility‐neutral under the studied protocols.

### 4.2. Tolerability/Acceptability Outcomes

#### 4.2.1. Pain and Discomfort

Across RCTs, vibration produced at most a small and inconsistent reduction in orthodontic pain. In fixed‐appliance studies, the characteristic postactivation trajectory—pain peaking at approximately 24 h followed by a gradual decline—remained unchanged. Any early reductions in pain were generally small, protocol‐dependent, and confined to isolated time points in certain low‐intensity or micropulse regimens [9]. Other trials reported no meaningful differences in pain trajectories or analgesic consumption compared with control groups [48].

In clear aligner therapy, high‐frequency daily vibration was associated with numerically lower pain scores during the first days after aligner changes in some studies. However, these effects were transient, not consistently reproduced across visits or regimens, and occurred against the background of the inherently moderate pain profile typical of aligners [47]. Accelerated change schedules using sham controls did not demonstrate a robust or sustained analgesic advantage [51, 52].

Heterogeneity in vibration parameters (frequency, magnitude, and exposure time), appliance systems, force delivery, and pain assessment instruments (VAS, NRS, and face scales), combined with small sample sizes and frequent industry involvement, further limits the generalizability of the findings. Several trials reported no benefit over initial alignment or longer treatment phases [49]. Overall, the available evidence does not support the routine prescription of vibration devices solely for pain control; any analgesic effect appears small, short‐lived, highly protocol‐specific, and based on low to very low certainty evidence.

#### 4.2.2. Oral Health–Related Quality of Life (OHIP‐14)

Evidence from Chouinard [15] and Katchooi et al. [51] indicates that adjunctive vibration does not significantly improve oral health–related quality of life, despite slightly lower OHIP‐14 scores in the vibration groups. The lack of statistical significance likely reflects low baseline OHIP‐14 values, leading to a floor effect and small sample sizes that reduce statistical power to detect modest differences.

Broader data suggest that appliance type (e.g., clear aligners versus fixed appliances) is the primary determinant of oral health–related quality of life, whereas adjunctive interventions such as vibration exert minimal influence and are overshadowed by stronger clinical and behavioral factors [66]. Collectively, the available evidence supports the conclusion that vibration does not confer a clinically meaningful benefit in oral health–related quality of life and should not be promoted primarily as a means to enhance patient‐reported well‐being.

#### 4.2.3. Analgesic Use

Findings from Miles and Fisher [16], Woodhouse et al. [48], and Bragassa [50] indicate that vibration does not reliably reduce analgesic consumption. Any reductions observed within the first 24 h were small, transient, and not consistently reproduced across trials. This pattern likely reflects the fact that medication use is driven primarily by peak pain during the first 1–2 days, as well as by patient expectations and clinician guidance, so that modest changes in mean pain scores do not necessarily translate into altered dosing behavior. Overall, any analgesic‐sparing effect of vibration appears minimal, short‐lived, clinically unreliable, and supported by very low certainty evidence.

### 4.3. Biological Response/Surrogate Markers

#### 4.3.1. Biomarkers

Across seven RCTs evaluating gingival crevicular fluid and salivary biomarkers [10, 14, 15, 43, 44, 47, 54], adjunctive vibration did not generate profiles consistent with harmful or chronic inflammation. Importantly, inflammatory and bone‐remodeling biomarkers are surrogate outcomes and do not, on their own, establish clinical harm. Transient increases in IL‐1β or PGE_2_ may reflect expected mechanotransduction and physiologic remodeling during orthodontic tooth movement. Therefore, in the absence of concordant clinical deterioration (e.g., increased root resorption, periodontal breakdown, or abnormal mobility), statistically significant biomarker shifts should be interpreted as biologic response signals rather than evidence of harm. Consistent with this interpretation, transient and site‐specific increase in mediators such as interleukin‐1β (IL‐1β) and prostaglandin E_2_ (PGE_2_) in response to vibratory stimuli are compatible with physiological bone remodeling and were not accompanied by adverse clinical end points, such as increased root resorption, periodontal breakdown, or abnormal tooth mobility.

In broader or longer‐term assessments, the RANKL/OPG ratio and multiplex cytokine panels showed minimal or no consistent differences relative to controls, with values remaining within the expected biological range for orthodontic tooth movement. Taken together, these data suggest that clinically used vibration protocols do not impose an unsafe inflammatory burden. Nonetheless, larger and longer‐duration studies are warranted to exclude subtle or cumulative subclinical effects, particularly in susceptible subgroups or under higher cumulative vibration exposure.

### 4.4. Limitations

This review has several methodological strengths, most notably the outcome‐level assessment of risk of bias for each prespecified safety variable rather than a single global judgment per study. This outcome‐based approach provides a more granular view of where methodological weaknesses lie and enhances the interpretability of safety findings across different end points.

However, important limitations remain. Most included trials were small and rated as having some concerns or high risk of bias, which reduces the certainty of effect estimates and limits the ability to detect uncommon or delayed adverse events. There was substantial clinical and methodological heterogeneity in orthodontic contexts, intervention protocols, outcome definitions, and follow‐up durations, which restricted the feasibility and robustness of quantitative synthesis.

Furthermore, many safety outcomes were secondary end points, assessed using different instruments and time points, and were often reported incompletely, with predominantly short‐term follow‐up. As a result, the findings should be interpreted with appropriate caution. Larger, well‐designed randomized trials with standardized, prospectively defined safety outcomes, longer follow‐up, and consistent reporting of harms are still needed to confirm the long‐term safety profile of VA‐OTM and to clarify whether specific protocols or patient subgroups might derive differential benefit or risk.

## 5. Conclusions

In terms of clinical safety end points, the RCTs assessing root resorption showed no consistent protective effect of vibration when evaluated by conventional radiographs, and the modest reduction observed in one experimental premolar‐tipping model remains of uncertain clinical relevance. Periodontal indices and tooth mobility (periotest) were generally similar between vibration and control groups, supporting short‐ to medium‐term periodontal and pulpal safety under the studied protocols.

Regarding tolerability/acceptability outcomes, this review indicates that adjunctive low‐intensity intraoral vibration, as delivered by the evaluated intraoral devices, does not provide a reliable or clinically meaningful reduction in orthodontic pain or analgesic use during canine retraction, alignment and leveling, en masse retraction, or clear aligner therapy. Any reported analgesic effects were small, protocol‐specific, and limited to isolated time points, with oral health–related quality of life (OHIP‐14) showing no consistent improvement over conventional treatment alone.

For biological response/surrogate markers, changes in gingival crevicular fluid and salivary biomarkers of inflammation and bone remodeling were heterogeneous and transient.

Nevertheless, the predominance of “some concerns” in risk of bias assessments, short follow‐up periods, heterogeneous devices and vibration parameters, and incomplete harms reporting mean that rare or delayed adverse effects cannot be excluded, and these results should not be extrapolated to untested devices or dosing schedules. Within the limitations of predominantly low to very low certainty evidence, the current evidence supports reassuring patients that the evaluated vibration protocols are unlikely to compromise root integrity, periodontal health, or tooth stability, but does not justify recommending vibration primarily for pain control, quality‐of‐life improvement, or root‐resorption prevention until larger, high‐quality trials with standardized safety outcomes are available.

**Nomenclature**
BOP: Bleeding on probing
CCTs: Controlled clinical trials
CRR: Crown–root ratio
GCF: Gingival crevicular fluid
GRADE: Grading of Recommendations Assessment, Development, and Evaluation
IL‐1β: Interleukin‐1 beta
McHarm: Modified McMaster University Harms scale
NRS: Numeric rating scale
OHIP‐14: Oral health impact profile‐14
OPG: Osteoprotegerin
PD: Probing depth
PGE_2_: Prostaglandin E_2_
PI: Plaque index
PICOS: Population, Intervention, Comparison, Outcomes, Study design
PRISMA: Preferred Reporting Items for Systematic Reviews and Meta‐Analyses
PROs: Patient‐reported outcomes
PTV: Periotest value
RANKL: Receptor activator of nuclear factor kappa‐B ligand
RCTs: Randomized controlled trials
RoB 2: Risk of bias 2 tool
RoB‐Var: Risk of bias by variables
UR1: Upper right central incisor
VA‐OTM: Vibration‐assisted orthodontic tooth movement
VAS: Visual analog scale.

## Author Contributions

Mohamad Radwan Sirri and Mohamad Yaman Salahi Alasbahi conducted the literature search, screened full‐text articles, and selected eligible studies for inclusion. Mohamad Radwan Sirri and Mohammad Osama Namera assessed the risk of bias using the RoB 2 tool, coordinated the tables, and managed the references. Mohamad Radwan Sirri, Mohamad Yaman Salahi Alasbahi, and Zaher Alswaidan wrote the initial drafts of the manuscript. Mohamad Yaman Salahi Alasbahi assisted in resolving conflicts during study selection and risk of bias assessment and contributed to the writing process. Mohammad Osama Namera, Mohamad Yaman Salahi Alasbahi, and Zaher Alswaidan helped formulate the focused review question, supervised various stages of the review, were involved in developing the concept of this review, and assisted with data analysis and interpretation of the results.

## Funding

No funding was received for this research.

## Disclosure

All authors have reviewed and approved the final version of the paper.

## Ethics Statement

The authors have nothing to report.

## Conflicts of Interest

The authors declare no conflicts of interest.

## Supporting Information

Additional supporting information can be found online in the Supporting Information section.

## Supporting information


**Supporting Information 1** Table S1: PRISMA‐Harms checklist.

**Supporting Information 2** Table S2: Electronic search strategy.

**Supporting Information 3** Table S3: The RoB 2 tool domains and judgments.

**Supporting Information 4** Table S4: Studies excluded and reasons for exclusion.

**Supporting Information 5** Table S5: Risk of bias of the included RCTs in this systematic review, with supporting reasons.

## Data Availability

The datasets used during the current review are available from the corresponding author upon reasonable request.
