# Supplementary material for: Safety Outcomes and Related Tolerability and Biological Responses of Vibration‐Assisted Orthodontic Tooth Movement: A Harm‐Focused Systematic Review of RCTs
Source: Int J Dent. 2026 Feb 18;2026:7774426. doi: 10.1155/ijod/7774426 (PMC12914218; doi:10.1155/ijod/7774426)
Supplement: Supplementary file 2 — Supporting Information 2 Table S2: Electronic search strategy. [file IJOD-2026-7774426-s002.docx]

| **Supplementary Table 2:** Electronic search strategy | | |
| --- | --- | --- |
| **No** | **Source** | **Search strategy (sets for this database)** |
| 1 | PubMed / MEDLINE | S1 (Population): (“Orthodontic Tooth Movement”[Mesh] OR “Tooth Movement Techniques”[Mesh] OR orthodontic*[tiab] OR “tooth movement”[tiab]) \nS2 (Intervention—Vibration): (“Vibration”[Mesh] OR vibration*[tiab] OR vibratory[tiab] OR “mechanical vibration”[tiab] OR “cyclic force*”[tiab] OR “high-frequency vibration”[tiab] OR HFV[tiab] OR “micropulse*”[tiab] OR AcceleDent[tiab] OR OrthoAccel[tiab]) \nS3 (Safety/harms & outcomes): (adverse OR “adverse event*” OR harm* OR safety OR “side effect*” OR pain[Mesh] OR pain[tiab] OR discomfort[tiab] OR “Analgesics”[Mesh] OR analgesic*[tiab] OR “Quality of Life”[Mesh] OR OHIP*[tiab] OR “Root Resorption”[Mesh] OR “root resorption”[tiab] OR “Periodontal Index”[Mesh] OR periodontal[tiab] OR “Tooth Mobility”[Mesh] OR “tooth mobility”[tiab] OR “Biomarkers”[Mesh] OR biomarker*[tiab]) \nS4 (Study design): (randomized OR randomised OR placebo OR sham OR “split-mouth” OR crossover OR “parallel group”) \nCombine: S1 AND S2 AND S3 AND S4. |
| 2 | Embase (Ovid) | S1: exp *tooth movement technique/* OR (orthodontic* OR “tooth movement”).ti,ab. \nS2: exp *vibration/* OR (vibration* OR vibratory OR “mechanical vibration” OR “cyclic force*” OR “high-frequency vibration” OR HFV OR micropulse* OR AcceleDent OR OrthoAccel).ti,ab. \nS3: (adverse OR harm* OR safety OR “side effect*”).ti,ab. OR exp *tooth pain/* OR discomfort.ti,ab. OR exp *analgesic agent/* OR exp *quality of life/* OR OHIP*.ti,ab. OR exp *root resorption/* OR exp *periodontal index/* OR exp *tooth mobility/* OR exp *biological marker/*. \nS4: (random*:ti,ab. OR placebo:ti,ab. OR sham:ti,ab. OR “split mouth”:ti,ab. OR crossover:ti,ab.) \nCombine: S1 AND S2 AND S3 AND S4. |
| 3 | Scopus | S1: TITLE-ABS-KEY(orthodontic* OR “tooth movement”) \nS2: TITLE-ABS-KEY(vibration* OR vibratory OR “mechanical vibration” OR “cyclic force*” OR “high-frequency vibration” OR HFV OR micropulse* OR AcceleDent OR OrthoAccel) \nS3: TITLE-ABS-KEY(adverse OR “adverse event*” OR harm* OR safety OR pain OR discomfort OR analgesic* OR OHIP* OR “root resorption” OR “periodontal index” OR “tooth mobility” OR biomarker*) \nS4: TITLE-ABS-KEY(random* OR placebo OR sham OR “split-mouth” OR crossover OR “parallel group”) \nCombine: S1 AND S2 AND S3 AND S4. |
| 4 | Web of Science Core Collection | S1: TS=(orthodontic* OR “tooth movement”) \nS2: TS=(vibration* OR vibratory OR “mechanical vibration” OR “cyclic force*” OR “high-frequency vibration” OR HFV OR micropulse* OR AcceleDent OR OrthoAccel) \nS3: TS=(adverse OR “adverse event*” OR harm* OR safety OR pain OR discomfort OR analgesic* OR OHIP* OR “root resorption” OR “periodontal index” OR “tooth mobility” OR biomarker*) \nS4: TS=(random* OR placebo OR sham OR “split-mouth” OR crossover OR “parallel group”) \nCombine: S1 AND S2 AND S3 AND S4. |
| 5 | Cochrane CENTRAL | S1: (orthodontic* OR “tooth movement”):ti,ab,kw \nS2: (vibration* OR vibratory OR “mechanical vibration” OR “cyclic force*” OR “high-frequency vibration” OR HFV OR micropulse* OR AcceleDent OR OrthoAccel):ti,ab,kw \nS3: (adverse OR “adverse event*” OR harm* OR safety OR pain OR discomfort OR analgesic* OR OHIP* OR “root resorption” OR “periodontal index” OR “tooth mobility” OR biomarker*):ti,ab,kw \nCombine: S1 AND S2 AND S3. |
| 6 | Trip Database | Query: (orthodontic* OR “tooth movement”) AND (vibration* OR vibratory OR “mechanical vibration” OR “cyclic force*” OR “high-frequency vibration” OR HFV OR micropulse* OR AcceleDent OR OrthoAccel) AND (adverse OR harms OR safety OR pain OR discomfort OR OHIP* OR analgesic* OR “root resorption” OR “periodontal index” OR “tooth mobility” OR biomarker*). |
| 7 | Google Scholar (grey literature discovery) | Query: “orthodontic tooth movement” AND (vibration OR vibratory OR “mechanical vibration” OR “cyclic force*” OR “high-frequency vibration” OR HFV OR micropulse* OR AcceleDent OR OrthoAccel) AND (adverse OR safety OR pain OR OHIP OR analgesic* OR “root resorption” OR “periodontal index” OR “tooth mobility” OR biomarker). |
