# Supplementary material for: Safety Outcomes and Related Tolerability and Biological Responses of Vibration‐Assisted Orthodontic Tooth Movement: A Harm‐Focused Systematic Review of RCTs
Source: Int J Dent. 2026 Feb 18;2026:7774426. doi: 10.1155/ijod/7774426 (PMC12914218; doi:10.1155/ijod/7774426)
Supplement: Supplementary file 3 — Supporting Information 3 Table S3: The RoB 2 tool domains and judgments. [file IJOD-2026-7774426-s003.docx]

| **Supplementary Table 3.** The RoB 2 tool domains and judgments | | |
| --- | --- | --- |
| **Domains** | **Judgments** | |
| **RoB 2.0 tool (For RCTs)** | | |
| 1. Bias arising from the randomization process | **overall** | **Low, Some**  **concerns, High** |
| 1. Bias due to deviations from intended interventions |  |  |
| 1. Bias due to missing outcome data |  |  |
|  |  |  |
| 1. Bias in measurement of the outcome |  |  |
| 1. Bias in the selection of the reported result |  |  |
| **RoB 2.0 tool: RCTs**: randomized clinical trials; **Low**: if all fields were estimated as "at low risk of bias"; **Some concerns**: if at least one domain was assessed as "some concerns" but not to be at "high risk of bias" for any domain; **High**: if at least one or more fields were estimated as "at high risk of bias" or if there were some concerns for multiple domains in a way that substantially lowered confidence in the result. | | |
