# Supplementary material for: Safety Outcomes and Related Tolerability and Biological Responses of Vibration‐Assisted Orthodontic Tooth Movement: A Harm‐Focused Systematic Review of RCTs
Source: Int J Dent. 2026 Feb 18;2026:7774426. doi: 10.1155/ijod/7774426 (PMC12914218; doi:10.1155/ijod/7774426)
Supplement: Supplementary file 4 — Supporting Information 4 Table S4: Studies excluded and reasons for exclusion. [file IJOD-2026-7774426-s004.docx]

| **Supplementary Table 4.** Studies excluded and reasons for exclusion | | |
| --- | --- | --- |
| **NO** | **Study** | **Reason for exclusion** |
| **1** | Farouk K, Shipley T, El-Bialy T; Effect of the application of high-frequency mechanical vibration on tooth length concurrent with orthodontic treatment using clear aligners: A retrospective study. J Orthod Sci 2018;7:20. doi: 10.4103/jos.JOS_53_18. | non-randomized  controlled trial |
| **2** | DiBiase AT, Woodhouse NR, Papageorgiou SN, et al.; Effects of supplemental vibrational force on space closure, treatment duration, and occlusal outcome: A multicenter randomized clinical trial. Am J Orthod Dentofacial Orthop 2018;153(4):469-480.e4. doi: https://doi.org/10.1016/j.ajodo.2017.10.021. | Inadequate description of materials, methods, treatment groups, interventions, or outcomes regarding root resorption |
| **3** | Lombardo L, Arreghini A, Huanca Ghislanzoni LT, et al.; Does low-frequency vibration have an effect on aligner treatment? A single-centre, randomized controlled trial. Eur J Orthod 2019;41(4):434-443. doi: 10.1093/ejo/cjy076. |  |
| **4** | Telatar BC, Gungor AY; Effectiveness of vibrational forces on orthodontic treatment : A randomized, controlled clinical trial. J Orofac Orthop 2021;82(5):288-294. doi: 10.1007/s00056-020-00257-z. |  |
| **5** | Yildiz O, Yagci A, Hashimli N; A different method to accelerate orthodontic tooth movement: Randomized controlled trial. Balk J Dent Med. 2023;27:51-55. doi: 10.5937/bjdm2301051Y. |  |
