# Supplementary material for: Safety Outcomes and Related Tolerability and Biological Responses of Vibration‐Assisted Orthodontic Tooth Movement: A Harm‐Focused Systematic Review of RCTs
Source: Int J Dent. 2026 Feb 18;2026:7774426. doi: 10.1155/ijod/7774426 (PMC12914218; doi:10.1155/ijod/7774426)
Supplement: Supplementary file 5 — Supporting Information 5 Table S5: Risk of bias of the included RCTs in this systematic review, with supporting reasons. [file IJOD-2026-7774426-s005.docx]

| **Supplementary Table 5.** Risk of bias of the included RCTs in this systematic review, with supporting reasons | | | | | | | |
| --- | --- | --- | --- | --- | --- | --- | --- |
| **Pain/Discomfort** | D1: Randomization process | D2: Deviations from intended interventions | D3: Missing outcome data | D4: Measurement of the outcome | D5: Selection of the reported result | **Overall Risk of Bias** |  |
| Abd ElMotaleb et al.  (2024) | Some concerns — Computer-generated 1:1 sequence (random.org); allocation concealment not described; baseline age balanced | Some concerns — Participants/orthodontist not blinded; AcceleDent adherence monitored (~20 min/day ≈4 months); knowledge of assignment could affect behavior | Low risk: Pain VAS at 0, 24 h, 48 h, 72 h, 7 d in week 1; no missing pain forms reported | Some concerns — Pain is self-reported, and participants knew whether they used the device (no sham), so expectation and awareness could influence ratings. | Trial preregistered (NCT05818527) with specified timepoints. | Some concerns |  |
| Alansari et al.  (2018) | Some concerns — Randomized, single-blinded multicenter with block randomization; allocation concealment not reported | High risk: Postrandomization exclusions for poor aligner/VPro5 compliance; sham arm discontinued; analysis appears per-protocol | Some concerns — Attrition across groups and discontinuation of sham; potential bias if missingness relates to compliance → Some concerns. | Low risk — A sham arm was used, providing participant blinding that minimizes expectation-driven distortion of self-reported pain. | Some concerns — No explicit preregistration for pain endpoints/timepoints | High risk |  |
| Azeem et al.  (2019) | Some concerns — Splitmouth; side randomized using random number tables by supervisor; allocation concealment not described | Some concerns — Participants unblinded to vibration side; adherence tracked via diary; expectations/cointerventions may influence behavior | Low risk: VAS diary daily in week 1, then weekly with reminders; no substantial missingness noted | Some concerns — Split-mouth without a true sham; participants could tell which side received vibration, so pain reporting may be influenced by awareness. | Some concerns — No trial registration/protocol for pain reporting was identified. | Some concerns |  |
| Bisht et al.  (2019) | Some concerns — Clinical study on vibration and pain/alignment is indicated by title/summary; sequence generation/concealment not described | Some concerns — Participants are likely unblinded to the vibration device; behavior/analgesic use may be influenced | Some concerns — Insufficient detail on pain-data completeness, without explicit response rates | Some concerns — No evidence of a sham; allocation likely obvious to participants, which can bias subjective pain scores. | Some concerns — No explicit preregistration/protocol for pain endpoints/timepoints | Some concerns |  |
| Bragassa  (2018) | Some concerns — Parallel randomized trial and described as “double-blinded,” but concealment is not detailed | Some concerns — Use vs no-use of vibration alongside accelerated schedules; participants likely aware despite attempts at blinding | Low risk: Multiple defined timepoints; no major pain missingness reported | Some concerns — Despite “blinding” language, a convincing participant sham isn’t demonstrated; device use is likely distinguishable to patients, risking biased self-reports. | Some concerns — No clear preregistration for pain/timepoints. | Some concerns |  |
| Bulic  (2017) | Some concerns — RCT; blinding for measurement described; allocation concealment not fully specified | Some concerns — Participants likely unblinded to AcceleDent use; adherence tracked | Some concerns — Flow reported and repeated surveys collected; withdrawals present | Some concerns — No clearly described sham; pain is participant-reported with aware participants, leaving room for expectation bias. | Some concerns — No explicit preregistration for pain outcomes/timepoints | Some concerns |  |
| Chouinard et al.  (2016) | Some concerns — Randomized clinical trial stated; concealment not detailed | Some concerns — The Vibration device makes participant blinding difficult | Some concerns — Pain as outcome not explicitly detailed here; absent specifics | Some concerns — No sham indicated; participants knew if they were using a vibration device, which can alter perceived pain. | Some concerns — No preregistration for pain/timepoints identified. | Some concerns |  |
| DiBiase et al.  (2016) | Low risk: Three-arm RCT with centrally generated random sequence and allocation concealment reported; baseline characteristics balanced; trial registration exists (EudraCT/ClinicalTrials.gov), though registration appears part-way through the overall recruitment period | Some concerns — Full blinding of patients/clinicians was not feasible across all arms (the fixed-appliance-only arm knew it had no device). Device compliance timing had technical limitations; adherence was monitored verbally. These factors could plausibly alter behavior/analgesic use and thus pain reporting → Some concerns | Low risk: Flow diagram shows few and balanced losses; pain data collected at planned time points; complete-case analysis used with no signal that missingness depended on true pain values | Low risk — Device arms included a sham to blind participants, reducing measurement bias in self-reported pain. | Some concerns — The trial was registered, and several methodological details are specified. There is no direct evidence of selective reporting for the pain outcome in this paper. However, there is no pre-published, detailed protocol/SAP that clearly prespecified the comparative pain analyses and time windows — so some uncertainty remains. | Some concerns |  |
| Kalemaj et al.  (2017) | Some concerns — 3-arm parallel RCT (1:1:1); concealment not reported | Some concerns — Participant blinding unlikely with vibration; standardized protocols across arms | Low risk: Reported no loss to follow-up | Some concerns — Three-arm design without a sham; participant awareness of intervention status can influence self-reported pain. | Some concerns — No explicit preregistration for pain endpoints/timepoints | Some concerns |  |
| Katchooi et al.  (2018) | Low risk: Block-stratified randomization by age/sex; list created in R; centralized assignment via a remote coordinator; triple-blinded (participants, investigators, assessors) → strong concealment & blinding | Low risk: True sham device (amplitude = 0) with IRB-approved deception to preserve blinding; compliance objectively downloaded + aligner wear checks → deviations unlikely | Low risk: CONSORT shows no loss to follow-up (one discontinuation due to device swap error); pain was collected via scheduled questionnaires | Low risk — True sham device (e.g., zero-amplitude) with robust participant blinding, minimizing expectancy effects on pain ratings. | Some concerns — Pre-registered (NCT02438280); protocol changes logged pre-start; no full published protocol text | Some concerns |  |
| Lobre et al.  (2016) | Low risk: Block randomization with sequence concealed from investigators; allocation by an independent designee using a computer-generated list | Some concerns — Participants not blinded to daily home device; NSAID restrictions and standardized instructions reduce — yet don’t remove — behavioral deviations. | Low risk: Dense pain schedule (7 time points in week 1, then weekly for 4 months; 40 data points/participant) with N≈70; no notable missingness reported | Some concerns — No sham; daily home device use makes allocation obvious to participants, risking expectation-biased pain reports. | Some concerns — No explicit pre-registration/protocol for pain timepoints. | Some concerns |  |
| Mayama et al.  (2022) | Low risk: Split-mouth, double-blind RCT with random side allocation; tooth movement measured blinded | Low risk: Chairside monthly vibration (3 min) under a standardized protocol; participants and assessors blinded to side | Low risk: CONSORT + final per-side N=23/23; no imbalance in pain data noted | Low risk — Double-blind split-mouth setup effectively masked the treated side, functioning as participant blinding for self-reported pain. | Some concerns — No clear registry ID stated; detailed methods without an explicit registration number. | Some concerns |  |
| Miles et al.  (2012) | Some concerns — RCT with block allocation; 66 patients; allocation concealment not detailed | Some concerns — Participants unblinded to Tooth Masseuse (home use 20 min/day); possible behavior/analgesic differences | Low risk: Pain was assessed at five time points over 10 weeks; no major missingness was reported | Some concerns — No sham; participants knew whether they used the Tooth Masseuse, which can sway subjective pain scores. | Some concerns — No pre-registration for pain endpoints/timepoints | Some concerns |  |
| Miles & Fisher  (2016) | Low risk: Single-blind with opaque sealed envelopes (permuted blocks); operator & assessor blinded, patients unblinded | Some concerns — Patient knowledge of allocation (home device) may influence behavior; standardized protocol & analgesic guidance mitigate but don’t eliminate | Low risk: No loss to follow-up; no missing data; reliability checks reported | Some concerns — Operator/assessor were blinded, but patients were not, and no sham was used, so participant-reported pain may be expectation-biased. | Some concerns — No explicit trial registration; outcomes unchanged after start (reported) | Some concerns |  |
| Pescheret (2017) | Some concerns — Described as a prospective randomized controlled study with ethics approval, the thesis text available does not detail random sequence generation or allocation concealment | High risk: Interventions differed not only by vibration device but also by aligner wear interval (e.g., 7-day vs 14-day changes). No participant blinding; potential incentives and protocol differences likely to affect behavior and pain perception | Some concerns — The accessible results sections do not clearly quantify attrition for pain assessments; no evidence that missingness was negligible or balanced | Some concerns — No sham and protocol differences (e.g., aligner change intervals) likely made allocation apparent, shaping pain ratings. | Some concerns — No prospective registration/protocol specifying pain endpoints and time windows; pain treated as a secondary outcome with multiple time points, allowing analytical flexibility | High risk |  |
| Taha et al.  (2019) | Some concerns — Small pilot RCT using simple randomization (e.g., GraphPad) described; allocation concealment not clarified; pilot sample size elevates uncertainty | Some concerns — Two-arm design (device vs none) without a sham; participants unblinded; device adherence modest (~56%) and declining. Behavior/co-interventions (e.g., analgesics) may differ by knowledge of allocation; ITT handling is not clearly stated | Low risk: Pain was reported at prespecified intervals over ~12 weeks; no major imbalance or dropout affecting pain reporting was indicated | Some concerns — Device vs no device with no sham; participants’ knowledge of allocation can influence subjective pain outcomes. | Some concerns — Pilot with IRB approval but no trial registration reported; multiple outcomes/time points raise selective reporting concerns for the pain domain. | Some concerns |  |
| Woodhouse et al. (2015) | Low risk — Three-arm parallel RCT with central sequence generation (GraphPad) and central allocation concealment independent of operators (“undertaken centrally at King’s College London, independently from the clinical operators, following recruitment”). Groups balanced at baseline. | Low risk — Interventions standardized (same brackets/archwires; identical sham device for the device arms). Participants with devices received identical instructions; analyses adjusted for covariates (incl. analgesic use). No evidence of deviations that would bias the effect-of-assignment estimate. | Low risk — Questionnaire return: 80/81 (99%) at T1, 77/81 (95%) at T2; missingness classified as MAR and not dependent on covariates. Minimal, balanced loss suggests little risk that missing data biases pain estimates. | Some concerns — Pain captured by 100-mm VAS at multiple time points with coded data and a blinded assessor/statistician; participants were blinded only between functional vs sham, but could not be blinded vs fixed-only (no device). Because pain is participant-reported, awareness of being in “fixed-only” may influence ratings | Low risk — Trial registered (EudraCT & ClinicalTrials.gov), primary outcome pre-specified (maximum pain), “no changes to outcomes” after commencement, and complete reporting (primary + secondary including analgesic use) with appropriate multiplicity notes (Bonferroni for secondary). | Some concerns |  |
| **Oral-health QoL (OHIP-14)** | D1: Randomization process | D2: Deviations from intended interventions | D3: Missing outcome data | D4: Measurement of the outcome | D5: Selection of the reported result | **Overall Risk of Bias** |  |
| Chouinard (2016) | Some concerns — Reported as an RCT, but allocation concealment is not clearly described; baseline procedures are standardized but lack detail on sequence generation and concealment | Some concerns — No sham; participants knew whether they used the vibration device. Although instructions and clinic protocols were standardized, knowledge of allocation could alter behavior and responses | Some concerns — OHIP-14 was collected and analyzed (e.g., non-parametric tests noted), but the paper does not quantify OHIP-14-specific missingness or demonstrate balance of any losses | Some concerns — OHIP-14 is described as a validated self-report instrument (14 items, 0–4), but participants were not blinded, so expectation/awareness can bias self-reported quality-of-life scores | Some concerns — No preregistered protocol/SAP specifying OHIP-14-time windows or primary contrasts is presented; with multiple outcomes/timepoints, some analytical flexibility remains | Some concerns |  |
| Katchooi et al. (2018) | Low risk: Block-stratified randomization (e.g., by age/sex) with centralized assignment; randomization list generated with software; procedures described with good clarity | Low risk: Triple blinding (participants, investigators, assessors) with a true sham device; device usage and aligner wear monitored, reducing allocation-related deviations | Low risk: CONSORT-style flow and repeated questionnaire collection with no signal of differential missingness for QoL data | Low risk: OHRQoL captured with validated questionnaires (e.g., OHIP-14); because participants were blinded via sham, measurement bias in self-reported QoL is minimized | Some concerns — Trial registered, but no fully pre-published protocol/SAP detailing OHIP-14 analysis (time windows, primary contrasts); some residual uncertainty about selective reporting remains | Some concerns |  |
| **Analgesic use** | D1: Randomization process | D2: Deviations from intended interventions | D3: Missing outcome data | D4: Measurement of the outcome | D5: Selection of the reported result | **Overall Risk of Bias** |  |
| Bragassa  (2018) | Some concerns — The thesis reports a “double-blinded parallel, randomized prospective clinical trial” with allocation into 3 groups (2-week wear, 4-day wear without vibration, 4-day wear with vibration). However, concealment and sequence-generation details are not fully described | Low risk: Both patients and investigators are described as double-blinded, and there is no indication of imbalanced protocol deviations affecting the analgesic outcome; compliance was monitored. | Some concerns — The analgesic outcome is presented as percent “YES to meds” at four timepoints in figures/tables, but the study does not clearly state numbers analyzed per timepoint for this outcome, nor methods for handling any missing survey responses — hence some concerns. | Low risk: Analgesic use was self-reported (yes/no) via survey; because the study states double blinding, knowledge of assignment was unlikely to influence self-report. | Some concerns — The thesis does not mention trial preregistration or a pre-specified analysis plan for the analgesic endpoint (tertiary measure). Selective reporting cannot be ruled out. | Some concerns. |  |
| DiBiase et al.  (2016) | Some concerns — The paper analyzes three groups (Accel, Accel-sham, Fixed-only). Randomization and allocation concealment procedures are not fully described in the OIIRR paper section where the analgesic covariate is presented; thus, some concerns. (Groups are shown, but method details are not specified in the excerpted methods/results.) | Low risk: Presence of a sham device arm reduces the risk of deviations due to awareness. No evidence of differential co-interventions affecting analgesic use; judged low risk. (Groups include Accel and Accel-sham alongside fixed-only.) | Some concerns — “Use of painkillers during alignment” is tabulated as n (%) by group, but handling of any missing diary/questionnaire entries isn’t described; hence, some concerns for potential unreported missingness. | Some concerns — Analgesic use appears patient-reported; two arms (Accel vs sham) likely blinded, but the fixed-only group could not be blinded to device use. Because the outcome is participant-reported and at least one arm is unblinded | Some concerns — No trial registration/SAP for this outcome is cited in the OIIRR paper; analgesic use is included as a covariate without pre-specification details | Some concerns. |  |
| Miles & Fisher  (2016) | Some concerns — The study is randomized with a CONSORT flow, but allocation concealment and sequence details are not fully reported in the text available | Low risk: No sham device; however, there is no evidence of deviations from intended interventions affecting the effect-of-assignment estimate, and all participants completed follow-up, so low risk. | Low risk: The paper explicitly states “all 40 … charts were returned”; analgesic users are tabulated by timepoint (Tables VIII–IX). | some concerns.: Analgesic use was self-reported, and participants were not blinded to having a device versus no device (“No appliance” control). Self-reported outcomes are likely influenced by awareness of the assignment. | No preregistration/SAP for the analgesic endpoint is reported; although the endpoint is clearly tabulated, selective reporting cannot be excluded. | Some concerns. |  |
| **Root resorption** | D1: Randomization process | D2: Deviations from intended interventions | D3: Missing outcome data | D4: Measurement of the outcome | D5: Selection of the reported result | **Overall Risk of Bias** |  |
| Abd ElMotaleb et al.  (2024) | Some concerns — Parallel 2-arm RCT with computer sequence and trial registration reported; outcome assessor masked, but allocation concealment procedures not clearly described. | Some concerns — Participants/clinicians not blinded; adherence encouraged and monitored, but lack of a sham could influence behavior. | Low risk — Pre/post CBCT datasets explicitly stated (18 pre, 18 post) for analysis, indicating minimal missingness. | Some concerns — Root resorption assessed on CBCT as linear crown-to-apex length with assessor masking; however, without a sham device, the measurement process could still be influenced per RoB 2 when knowledge of intervention is possible. | Low risk — Prospectively registered (NCT05818527) and authors state no changes after trial commencement. | Some concerns. |  |
| DiBiase et al.  (2016) | Low risk — Central randomization, adequate sequence generation and allocation concealment; preregistered (EudraCT, ClinicalTrials.gov). | Low risk — Three-arm design included a sham device identical in appearance; participants and assessors were effectively blinded to functional vs sham, minimizing deviations. | Low risk — 9/81 excluded, evenly distributed; missingness judged MAR; complete-case analysis described. | Low risk — OIIRR measured on standardized long-cone periapicals, blinded assessor, image-magnification correction, and repeatability checks. | Low risk — Registered with stated outcomes and no changes post-commencement. | Low risk |  |
| Grove  (2011; thesis) | Some concerns — Split-mouth with random side assignment is shown, but sequence generation/concealment procedures are insufficiently detailed in the thesis appendices. | Some concerns — No sham; participant awareness of vibration vs no vibration possible in a split-mouth design; potential for performance deviations cannot be excluded. | Some concerns — Small sample; thesis does not clearly report attrition handling for extracted-tooth analyses. (Design and flow described, but completeness not explicitly quantified.) | Some concerns — Objective micro-CT crater volume is strong, but assessor blinding is not explicitly stated and no sham; thus, RoB 2 suggests caution re: measurement influenced by intervention knowledge. | Some concerns — Academic thesis with no preregistered protocol identified. (Methods detailed; registration absent.) | Some concerns. |  |
| Mayama et al.  (2022) | Some concerns — Randomization/concealment for groups not clearly detailed in the accessible sections; registered status not explicitly shown in methods excerpt. | Some concerns — Comparison of TM vs TM+V without a sham; blinding unlikely; potential behavior differences cannot be ruled out. | Low risk — Outcomes (pain; crown/root ratio) are presented with pre/post data; no missingness flagged in the results figures for the root metric. | Some concerns — Root resorption proxied by crown/root ratio pre- vs post-retraction; objective figure reporting but no sham, and assessor blinding not stated. | Some concerns — Reporting aligns with figures, but the prospective protocol/specification for this outcome is not explicitly shown in the excerpts. | Some concerns |  |
| Yilmaz et al.  (2021) | Low risk — Split-mouth random side assignment; trial registered (NCT04686617); outcome assessments blinded. | Some concerns — No sham device; although split-mouth reduces confounding and assessor blinding is reported, participant behavior could still differ between sides. | Low risk — All premolars extracted and scanned; no missingness indicated in methods/results. | Some concerns — Micro-CT crater volume measured with Fiji/ImageJ and SkyScan; assessor blinding stated, but no sham per your rule for Domain 4. | Low risk — Trial registration provided within the article front matter. | Some concerns. |  |
| **Biomarkers** | D1: Randomization process | D2: Deviations from intended interventions | D3: Missing outcome data | D4: Measurement of the outcome | D5: Selection of the reported result | **Overall Risk of Bias** |  |
| Alansari et al.  (2018) | Some concerns — Parallel groups with “Control”, “Sham”, and “HFA/VPro5” arms; allocation method not explicitly described; baseline table presented, and groups appear similar | Low risk: Protocolized use of VPro5 (120 Hz, 0.03 g) with dismissal of poor compliers; no evidence of differential co-interventions; analysis focuses on pre-specified outcomes. | Low risk: Flow diagram shows near-complete outcome capture; exclusions due to nontracking specified a priori; GCF successfully collected baseline and at the end of tray 2. | Low risk: Biomarkers measured from GCF using MILLI-Plex/Luminex; sampling steps, volumes, and processing details; laboratory assessors effectively blinded by coded plates; objective assay. | Some concerns — Trial outcomes reported for pain and GCF proteins; trial registration is not the driver here — reporting domains appear complete, but multiplicity across many markers is not clearly pre-specified in the article text. | Some concerns |  |
| Chouinard et al.  (2016) | Some concerns — Randomization mentioned in the parent trial context, but specific allocation concealment is not detailed in the biomarker sub-analysis | Low risk: Standardized orthodontic mechanics; no deviations reported that would differentially affect biomarker sampling. | Low risk: No substantial loss for the biomarker timepoints reported for the subset. | Low risk: Outcome measured objectively (ELISA/multiplex per study’s methods) with lab analysis blinded/coded as per center standards | Some concerns — Biomarker panel and timepoints are reported as planned for the study; however, lack of a public, detailed biomarker-analysis plan in the article text. | Some concerns |  |
| Gujar et al.  (2023) | Some concerns — “Simple randomization” to 125 Hz, 150 Hz, or control is stated, but sequence generation/allocation concealment not described; small sample size (n=30) | Low risk: Standardized retraction mechanics; instruction-based home use of a custom electric toothbrush; no evidence of systematic deviations. | Some concerns — All 30 enrolled analyzed across T0–T3 are implied; authors acknowledge limitations but do not show material missingness at biomarker timepoints → some concerns (unclear handling if any samples insufficient). | Some concerns — GCF PGE₂ collected with a microcapillary pipette; field collection is sensitive to saliva/plaque contamination (acknowledged by authors), and no explicit lab blinding is described | Some concerns — No prior analysis plan for PGE₂ multiplicity; conclusions emphasize significant differences; selective emphasis cannot be excluded | Some concerns |  |
| Kalemaj et al.  (2017) | Some concerns — Parallel groups with a vibration arm (AcceleDent) vs other; no details on sequence generation or concealment | Low risk: Use a schedule for standardized vibratory device (20 min/day for 4 weeks); no evidence of differential co-interventions. | Low risk: Follow-up for first 3 months with planned casts/GCF sessions; no notable differential attrition in the biomarker sampling reported | Some concerns — GCF IL-1β measured via ELISA from PerioPaper with Periotron quantification; operator-collected samples; lab blinding not stated | Some concerns — Biomarker (IL-1β) was specified as a main outcome, but no accessible pre-analysis plan; multiplicity across visits without adjustment was described | Some concerns |  |
| Leethanakul et al.  (2016) | Some concerns — Split-mouth with random allocation of the experimental side; concealment not described; small n=15. | Low risk: Vibration (125 Hz electric toothbrush) protocolized; contralateral control provides within-patient control; no deviations reported | Low risk: Monthly sampling across 3 months; no differential attrition reported; per-protocol style but complete pairs generally available | Some concerns — GCF IL-1β via ELISA (duplicate sites) with Periotron volume; operator not blinded at chairside; lab-blinding not explicitly stated | Some concerns — Outcomes (IL-1β and movement) were prespecified in the aims, but no published analysis plan; risk of selective emphasis remains. | Some concerns |  |
| Reiss et al.  (2020) | Low risk: Block randomization with opaque envelopes; single-blind; registered; ITT planned. | Low risk: Manufacturer-specified daily AcceleDent use; standardized fixed-appliance care; analyses conducted blinded; no deviations affecting saliva outcomes. | Low risk: 40 randomized with serial saliva at T0–T3; outcomes analyzed ITT; no differential missingness that would bias biomarkers | Low risk: Salivary multiplex panel (17 biomarkers) with standardized collection (timing to control circadian effects) and lab processing; assessors blinded by coding. | Low risk: Registered (ClinicalTrials.gov NCT02119455) with outcomes/timepoints; article reports full biomarker panel and RMAA as per plan | Low risk |  |
| Siriphan et al.  (2019) | Low risk: Parallel-group RCT (n=60) with three arms; randomization clearly described; adequate groups. | Low risk: Vibration delivered initially by investigator (days 1–7) then at home; control of orthodontic force at 60 cN; no deviations affecting GCF planned | Low risk: Serial GCF timepoints (T1–T4) with analysis of all randomized; no important missingness reported. | Some concerns — GCF RANKL/OPG by ELISA (PerioPaper/Periotron); subjects and first investigator not blinded to allocation; data analyst blinded → some concerns (performance/detection for biomarker could be influenced at chairside). | Low risk: Outcomes and timepoints prespecified; full null findings reported; selective reporting unlikely | Some concerns |  |
| **Periodontal indices** | D1: Randomization process | D2: Deviations from intended interventions | D3: Missing outcome data | D4: Measurement of the outcome | D5: Selection of the reported result | **Overall Risk of Bias** |  |
| Azeem et al.  (2019) | Some concerns — Split-mouth; the vibration side was randomly selected using random number tables. Allocation concealment is not described; randomization occurred after R1 within participants. Baseline hygiene normalized across subjects (scaling/polishing; inclusion required PI <10%), but the lack of concealment detail leaves some uncertainty. | Low risk — Oral hygiene was standardized (pre-bond supragingival scaling/polishing; hygiene instructions; monthly reminders). Participants were told not to use the powered brush for cleaning, only for vibration — reducing behavior differences that could bias PI. Split-mouth further limits confounding. No protocol deviations were reported that differentially affected PI. | Low risk — PI was recorded at four periods (R0–R3) on six sites per tooth; no PI-specific attrition is reported, and results show complete serial PI summaries (Table 3; p=1.000 at each time). | Low risk — Plaque Index (PI) scoring scheme is explicitly defined (0–3) and was performed by a blinded examiner; multi-site, multi-tooth assessment at each time point. Objective scoring with examiner blinding minimizes detection bias for periodontal indices. | Some concerns — PI is a secondary clinical outcome with multiple timepoints; no public, detailed pre-analysis plan specifying primary time windows or handling of multiplicity for periodontal indices. Although PI is fully presented, selective emphasis across timepoints cannot be fully excluded. | Some concerns |  |
| Kalemaj et al.  (2017) | Low risk — Three-arm parallel RCT with block randomization (variable block sizes) and centralized assignment independent of treating staff; CONSORT-style reporting; baseline comparability described. These details support robust sequence generation and concealment. | Low risk — Orthodontic mechanics (archwire sequence) and hygiene were standardized, with periodontal status screened/maintained (modified sulcus bleeding index <20% before, target <25% during treatment). No sham device, but for periodontal indices specifically, standardized hygiene control reduces the chance that awareness of assignment materially altered PI/PD. | Low risk — Follow-up across the first 3 months is clearly described; only minimal missing data are reported (mainly for GCF at a few timepoints). There’s no indication of PD/periodontal-index-specific missingness that would bias the outcome. | Some concerns — Periodontal measures reported include GCF quantity and PD. The paper states blinded assessment for models/samples/questionnaires via coding; however, chairside PD probing is typically operator-dependent and not explicitly confirmed as blinded at the time of examination. PD is objective, but potential awareness of allocation at chairside leaves a small detection-bias risk | Some concerns — Periodontal indices are secondary outcomes with repeated measures; although outcomes/timepoints are reported, there is no public, detailed biomarker/periodontal SAP specifying hierarchical endpoints or multiplicity handling for PD/GCF quantity, leaving room for analytical flexibility. | Some concerns |  |
| **Tooth mobility** | D1: Randomization process | D2: Deviations from intended interventions | D3: Missing outcome data | D4: Measurement of the outcome | D5: Selection of the reported result | **Overall Risk of Bias** |  |
| Chouinard et al.  (2016) | Some concerns — Block randomization with sex-stratified opaque envelopes is described, but key safeguards for concealment (e.g., sequential numbering, tamper-proof sealing, opened only after enrollment) are not fully reported, so adequacy of allocation concealment is uncertain. | Some concerns — Participants knew their allocation (no sham device; patients opened envelopes and received device instructions), so behavior/compliance could plausibly differ between arms despite standardized mechanics — thus some concerns for the effect-of-assignment estimand. | Low risk: Periotest mobility was scheduled and reported at all timepoints (T0–T3) with consistent group Ns and no signs that missingness depended on true mobility values. | Some concerns — Periotest is used with a standardized technique (multiple taps, averaging, positioning protocol). Still, the assessor was not explicitly stated as blinded for this outcome (operator dependence remains), so some concerns for detection bias. | Some concerns — Mobility was pre-listed and fully tabulated, but no pre-published protocol/SAP specified primary contrasts or multiplicity handling for mobility, leaving room for selective emphasis. | Some concerns |  |
